# Longitudinal resting-state EEG in amyloid-positive patients along the Alzheimer’s disease continuum: considerations for clinical trials

**DOI:** 10.1186/s13195-023-01327-1

**Published:** 2023-10-19

**Authors:** Elliz P. Scheijbeler, Willem de Haan, Cornelis J. Stam, Jos W. R. Twisk, Alida A. Gouw

**Affiliations:** 1 Clinical Neurophysiology and MEG Center, Neurology, Vrije Universiteit Amsterdam, Amsterdam UMC Location VUmc, Amsterdam, Netherlands; 2 Amsterdam Neuroscience, Neurodegeneration, Amsterdam, Netherlands; 3 Alzheimer Center Amsterdam, Neurology, Vrije Universiteit Amsterdam, Amsterdam UMC Location VUmc, Amsterdam, Netherlands; 4 Department of Epidemiology and Biostatistics, Vrije Universiteit Amsterdam, Amsterdam UMC Location VUmc, Amsterdam, Netherlands

**Keywords:** Longitudinal, Resting state, Electroencephalography, Alzheimer’s disease, Clinical trial design, Functional endpoint, Sample size

## Abstract

**Background:**

To enable successful inclusion of electroencephalography (EEG) outcome measures in Alzheimer’s disease (AD) clinical trials, we retrospectively mapped the progression of resting-state EEG measures over time in amyloid-positive patients with mild cognitive impairment (MCI) or dementia due to AD.

**Methods:**

Resting-state 21-channel EEG was recorded in 148 amyloid-positive AD patients (MCI, *n* = 88; dementia due to AD, *n* = 60). Two or more EEG recordings were available for all subjects. We computed whole-brain and regional relative power (i.e., *theta* (4-8 Hz), *alpha1* (8-10 Hz), *alpha2* (10-13 Hz), *beta* (13-30 Hz)), peak frequency, signal variability (i.e., *theta* permutation entropy), and functional connectivity values (i.e., *alpha* and *beta* corrected amplitude envelope correlation, *theta* phase lag index, weighted symbolic mutual information, inverted joint permutation entropy). Whole-group linear mixed effects models were used to model the development of EEG measures over time. Group-wise analysis was performed to investigate potential differences in change trajectories between the MCI and dementia subgroups. Finally, we estimated the minimum sample size required to detect different treatment effects (i.e., 50% less deterioration, stabilization, or 50% improvement) on the development of EEG measures over time, in hypothetical clinical trials of 1- or 2-year duration.

**Results:**

Whole-group analysis revealed significant regional and global oscillatory slowing over time (i.e., increased relative *theta* power, decreased *beta* power), with strongest effects for temporal and parieto-occipital regions. Disease severity at baseline influenced the EEG measures’ rates of change, with fastest deterioration reported in MCI patients. Only AD dementia patients displayed a significant decrease of the parieto-occipital peak frequency and *theta* signal variability over time. We estimate that 2-year trials, focusing on amyloid-positive MCI patients, require 36 subjects per arm (2 arms, 1:1 randomization, 80% power) to detect a stabilizing treatment effect on temporal relative *theta* power.

**Conclusions:**

Resting-state EEG measures could facilitate early detection of treatment effects on neuronal function in AD patients. Their sensitivity depends on the region-of-interest and disease severity of the study population. Conventional spectral measures, particularly recorded from temporal regions, present sensitive AD treatment monitoring markers.

**Supplementary Information:**

The online version contains supplementary material available at 10.1186/s13195-023-01327-1.

## Background

Alzheimer’s disease (AD) is diagnosed in vivo by abnormalities on core biomarkers, including amyloid-beta (Aβ) deposition, pathologic tau, and neurodegeneration. The disease is a continuum, spanning preclinical, mild cognitive impairment (MCI), and dementia stages [1]. The worldwide population of AD dementia patients is anticipated to exceed 150 million by 2050, unless means of delay, prevention, or treatment are found [2]. Current experimental treatments of AD revolve around reversing existing pathology, primarily focusing on Aβ removal. Aducanumab (ADUHELM™) and lecanemab (Leqembi™) were recently approved for treatment of AD in the US, with evidence of cognitive efficacy to be confirmed in post-marketing trials. Other promising anti-amyloid antibodies are currently awaiting FDA approval (donanemab) or are under phase 3 investigation (gantenerumab). The advent of these agents is accompanied by that of non-pharmacological interventions, such as magnetic stimulation of the precuneus [3].

A critical step in designing valid and useful AD clinical trials is selecting appropriate outcome measures. Clinical efficacy of an intervention is typically evaluated by an assessment of cognition and everyday functioning, with growing interest for cognitive and functional composite scores [4, 5]. Identifying a successful therapy using these measures is difficult in preclinical stages of AD, in which the target population has not yet demonstrated cognitive decline and may not do so in the near future. Biomarkers provide a way to evaluate pathologic processes, or biological responses to a therapeutic intervention, prior to their clinical presentation. Trials that use biomarkers to establish a drug-placebo difference are generally much shorter and smaller than trials that require demonstration of clinical benefit, due to the low sensitivity of cognitive measures [6]. While established in vivo fluid and neuroimaging AD biomarkers mirror molecular and structural brain changes associated with the disease [7], high costs and/or invasiveness make them less suited for serial measurements. Electroencephalography (EEG) biomarkers have been proposed as alternative measures to demonstrate the efficacy of novel therapeutics [8, 9]. The non-invasive, low-cost neurophysiological technique provides a relatively direct measure of neuronal activity and synaptic function. The temporal resolution of EEG allows for investigation of cortical rhythms during a resting-state condition, as well as of quick positive and negative voltage peaks in response to cognitive-motor events (i.e., event-related potentials). Resting-state EEG has the practical advantage that it does not require any response by a patient. This allows more severely impaired patients, who may not be able to perform tasks accurately, to be studied.

Different types of resting-state EEG measures have been used to quantify neurophysiological dysfunction in AD patients. These can roughly be divided into (i) spectral, (ii) functional connectivity, and (iii) entropy measures, according to the analytical methodology that is employed for computation. Increased relative *theta* power has been reported as early as the preclinical stage of AD [10, 11]. Enhanced relative *delta* power, as well as posterior relative *alpha* and *beta* power reduction, have repeatedly been reported in later stages of the disease [12–14]. Disrupted communication between brain regions is another well-established finding in AD. MCI and AD dementia patients show large-scale disruptions in functional connections (e.g., a loss of connectivity in *alpha* and *beta* frequency bands, increased *theta* band connectivity [15–17]) and selective vulnerability of cortical hub regions (i.e., highly connected nodes with a central position in the overall organization of a network [16, 18–21]). Growing evidence furthermore suggests a significant, progressive loss of entropy of neural activity over the course of the disease [22–25].

The degree of EEG abnormalities has been shown to correlate strongly with cognitive impairment in AD patients, in both cross-sectional [26] and longitudinal studies [27]. Several measures, including relative power [11, 28], the spectral power ratio (*delta/theta* + *alpha/beta* power) [29], spectral coherence [30], and complexity [31], have shown significant associations with neuropsychological measures. EEG measures have also been shown to be predictive of clinical progression, predicting conversion from the preclinical to MCI, or from the MCI to dementia stage of AD [10, 11, 32]. This association with cognitive and clinical assessments is part of what makes EEG measures an interesting target for treatment monitoring.

In the relatively short time span of a clinical trial, EEG measures are hypothesized to be more sensitive to change than biomarkers obtained from cerebrospinal fluid (CSF), positron emission tomography (PET), and magnetic resonance imaging (MRI). Using them as markers of target engagement could therefore potentially reduce the size, duration, and costs of clinical trials. Furthermore, as the availability of clinically efficacious medication grows, investigation of its impact on brain function will become of increasing interest. EEG measures provide an opportunity to study the neurophysiological mechanisms underlying therapeutic outcomes. At present, the main clinical goal of AD clinical trials is a slowing or halting of cognitive decline. Assessment of (stabilizing) treatment effects using EEG measures depends on deterioration of electrophysiological measures in the placebo group. Sample size estimates and power analyses are generally based on information from previous research. Available longitudinal EEG studies in AD patients however often lack statistical power due to the use of small cohorts [33, 34], do not make use of (recently) established diagnostic guidelines (e.g., include amyloid-negative patients) [32, 33, 35–38], or only report on a limited number of EEG measures [39]. A solid understanding of the natural course of the EEG in amyloid-positive patients along the AD continuum is needed to help improve clinical trial design and facilitate selection of the most suitable neurophysiological markers for trial implementation.

We modeled the development of a variety of resting-state EEG measures over time in a large, amyloid-positive AD cohort, including patients with MCI or dementia (*N* = 148). Faster rates of change were expected for regional (i.e., temporal and parieto-occipital) than whole-brain measures, considering that disruption of local neuronal activity precedes the emergence of whole-brain abnormalities. Spectral measures, particularly relative *theta* power, were expected to be most sensitive to change. The development of EEG measures over time was compared between groups stratified based on baseline disease stage (MCI or dementia) to investigate the effect of clinical disease severity on the measures’ rates of change. Fastest deterioration was expected in non-demented patients, as the presence of extensive structural brain changes in AD dementia patients may cause the rate of functional decline to slow down or plateau. We computed yearly and two-yearly effect sizes for a subset of EEG measures that showed significant deterioration over time. These values were used to estimate the minimum sample size required to detect different treatment effects (i.e., 50% less deterioration, stabilization, or 50% improvement) on the development of EEG measures over time, in hypothetical clinical trials of 1- or 2-year duration.

## Methods

### Study design and participants

We retrospectively included patients who had been evaluated and followed up in the memory clinic of the Amsterdam UMC Alzheimer center, or who participated in a multicenter AD clinical trial with central EEG analysis at the Amsterdam UMC EEGlab, between October 15, 2003, and January 1, 2019. All participants provided written informed consent for the use of their data for research purposes. Although AD represents a seamless disease continuum, patients can be assigned to progressive phases based on physical, cognitive, and functional assessments [1]. We differentiated between patients with MCI and dementia due to AD based on established clinical guidelines [40]. For a detailed description of all investigations that were performed as part of our routine diagnostic screening, see Van der Flier et al. [41]. Two or more EEG recordings were available for all participants. Recordings that were heavily contaminated with artifacts were excluded from analysis. Follow-up durations shorter than 3 months or longer than 3 years are not commonly employed in AD clinical trials. We therefore only evaluated follow-up recordings that were obtained within this time-frame. All participants were positive for Aβ deposition, as assessed using CSF Aβ42 (cut-off < 813 pg/ml, Tijms et al. (2018)) [42] or [^11^C] PiB amyloid PET investigation (the routine PET protocol has been described elsewhere [43, 44]). Tau pathology and neurodegeneration were characterized at baseline using CSF p-tau (cut-off > 52 pg/ml) and t-tau levels (cut-off > 375 pg/ml) [45]. Tau and neurodegeneration positive and negative patients (T +/- , N +/-) were included in this study. Medial temporal lobe atrophy (MTA), ranging from 0 (no atrophy) to 4 (severe atrophy), was rated on coronal T1-weighted MRI images. To evaluate the potential effect of pharmacological agents (i.e., cholinesterase inhibitors, anti-depressants, anti-epileptic drugs, anti-psychotics, benzodiazepines) on our findings, medication use was evaluated and scored.

### EEG acquisition and pre-processing

Twenty minutes eyes-closed resting-state EEG data was recorded on digital EEG systems from 21 electrodes at the positions of the 10–20 system: Fp2, Fp1, F8, F7, F4, F3, A2, A1, T4, T3, C4, C3, T6, T5, P4, P3, O2, O1, Fz, Cz, Pz. A common or average reference (including all electrodes except Fp2/1 and A2/1) was used. Electrode impedance was kept below 5 kΩ. Sample frequency (200, 250, 256, 500, or 512 Hz) and online filter settings (high-pass 0.16 or 0.5, low-pass 70 Hz) varied between clinical- and trial-related recording protocols. During acquisition, patients and their recordings were monitored by an EEG technician, in order to minimize artifacts and prevent drowsiness. Ten 4096-sample (sample frequency of 500 or 512 Hz) or 2048-sample (sample frequency of 200, 250, or 256 Hz) epochs of eyes-closed artifact-free data (containing no eye blinks, muscle artifact, slow eye movements, or EKG-artifacts) were selected from each EEG recording based on visual inspection of the data by one of the authors (E.S.).

### Analysis and outcome measures

Different types of measures were computed from the EEG waveforms, using open access *Brainwave* software (version 0.9.163.26, developed by Professor C.J. Stam, http://home.kpn.nl/stam7883/brainwave.html).

#### Spectral measures

For each of the 21 electrodes, the relative contribution (i.e., power) of different frequency bands (i.e., *theta* (4–8 Hz), *alpha1* (8–10 Hz), *alpha2* (10–13 Hz), and *beta* (13–30 Hz)) to the broadband EEG signal (0.5–48 Hz) were calculated using a fast Fourier transform. *Delta* (0.5–4 Hz) and *gamma* (30–48 Hz) frequencies were excluded from analysis because of their respective sensitivity to ocular [46] and electromyogenic artifacts [47, 48]. In addition, the peak frequency of the power spectrum was identified for each electrode as the median frequency between 4 and 13 Hz.

#### Functional connectivity measures

Functional connectivity refers to the statistical dependence, or ‘inter-relatedness’, between time series of electrophysiological activity in distinct regions of the brain. We estimated connectivity strength using different techniques:

The *amplitude envelope correlation* (AEC) [49–51] is a measure of amplitude-based connectivity between two time series. The linear correlation coefficient between the power envelopes of two time series was computed and normalized between 0 and 1, with 0.5 indicating no functional connectivity. To correct for the effects of volume conduction, we made use of pairwise orthogonalization in two directions (i.e., X to Y and Y to X) prior to AEC estimation [15, 49]. The AEC values (i.e., the correlation between the orthogonalized envelopes) for both directions were averaged, resulting in corrected AEC (AEC-c) values.

The *phase lag index* (PLI) [52] provides an estimate of phase-based connectivity between two time series. If no phase synchronization exists between two time series, the distribution of their phase differences is expected to be flat. Any deviation from this flat distribution indicates phase synchronization. The PLI is an asymmetry index for this distribution. Its values range between 0 and 1, with 0 indicating no connectivity and 1 referring to perfect phase locking. The measure is invariant against the presence of common sources (i.e., volume conduction), as it discards phase differences that center around 0 mod π.

A single AEC-c and PLI value was obtained for each of the 21 electrodes, by averaging over its 20 pair-wise connectivity values. This value indicates the average connectivity strength between that electrode and the rest of the brain. This corresponds to the notion of “weighted degree” or “node strength” in graph theory.

#### Entropy measures

### Single channel

Variability of each EEG time series was quantified using the *permutation entropy* (PE) [53]. The continuous EEG signal recorded from each electrode was transformed into a sequence of discrete symbols. Next, the Shannon’s information entropy of the symbol probability distribution was computed. High entropy values indicate a flat or uniform symbol probability distribution (i.e., high signal variability), whereas low entropy values indicate a more bell-shaped curve (i.e., low signal variability).

### Functional connectivity

The *weighted symbolic mutual information* (wSMI) [54] evaluates the extent to which two time series present nonrandom joint fluctuations. To quantify the information shared between two time series, the continuous signals are first transformed into sequences of discrete symbols (as is done to compute the PE). The joint probability of each pair of symbols gives the SMI, with high values indicating strong coupling. To correct for potential common-source artifacts, the weights of pairs of identical or opposite-sign symbols in the joint probability matrix are set to zero.

The *joint permutation entropy* (JPE) [55] integrates information on local signal variability (as reflected by the PE) and interregional coupling (as reflected by the wSMI). Again, the continuous signals are transformed into sequences of discrete symbols. This time, connectivity is defined as the Shannon’s information entropy of the joint probability matrix. To facilitate comparison to more conventional connectivity measures, we report inverted JPE (JPE_inv_) values, so that higher values correspond to stronger coupling. Note that measures based on a symbolic representation of the EEG time series (i.e., PE, wSMI and JPE_inv)_ require the choice of parameter settings, such as symbol size *n* and time-delay *τ*. In the present study, we made use of *n* = 4 and *τ* = 1. For more information on the role of these parameters in entropy computations and validation of the selected settings, see Scheijbeler et al. [55].

The investigated EEG outcome measures are presented in Table 1. We computed global (i.e., whole-brain) and regional averages (i.e., temporal—A2, A2, T4, T3, T6, T5 and parieto-occipital – P4, P3, O2, O1, Pz) for relative power, PE, AEC-c, PLI, wSMI, and JPE_inv_. The peak frequency of the power spectrum was computed over parieto-occipital sensors only. The AEC-c was computed in the *alpha* and *beta* band and the PLI in the *theta* band because of the reproducibility of these metrics infinding differences between AD dementia patients and cognitively healthy controls [15, 56]. The PE, wSMI, and JPE_inv_ were computed in the *theta* band [55], resulting in 31 measures of interest.

**Table 1:** EEG measures of interest

Region	Measure	Frequency band
Global, parieto-occipital, temporal	Relative power	Theta
		Alpha1
		Alpha2
		Beta
	AEC-c	Alpha
		Beta
	PLI	Theta
	PE	Theta
	wSMI	Theta
	JPE_inv_	Theta
Parieto-occipital	Peak frequency	-

*PE* Permutation entropy, *AEC-c* Corrected amplitude envelope correlation, *PLI* Phase lag index, *wSMI* Weighted symbolic mutual information, *JPE*_*inv*_ Inverted joint permutation entropy

### Statistical analysis

#### Group differences of demographic variables

Group differences for each demographic variable at baseline were assessed between the MCI and AD dementia subgroups using *χ*2 tests for categorical variables and Kruskal–Wallis tests for continuous variables.

#### Development of EEG measures over time

Development over time was analyzed using linear mixed models (LMMs). A LMM adjusts for the dependency of the repeated observations within a subject by modelling variability among individuals and including both fixed and random effects. The simplest form of a LMM only allows the intercepts to vary across subjects. In addition to a random intercept, it is possible that development over time varies across individuals, as reflected by a random slope. Model fit of “random intercept” and “random intercept and slope” models was compared using likelihood ratio tests. The best, or, in the case of similar fit, the simplest model, was used to analyze the development over time for the outcome variables of interest.

We first modeled the linear development of EEG measures over time within the whole cohort. *Time,* our covariate of interest, was included as a continuous variable in months. Its values reflect follow-up time between EEG recordings and were therefore unequally spaced in time. A separate LMM was built for each EEG outcome measure (Table 1*, n* = 31).

**Table 2:** Baseline demographic and clinical characteristics

	**MCI**	**AD dementia**	**Total**
***N***	88	60	148
Age, years (mean, SD)	70.7 (7.2)^**^	66.9 (8.2)	69.2 (7.8)
Sex (male/female)	46/42	31/29	77/71
MMSE (median, IQR)	27 (3)^***^	24 (5)	26 (4)
MTA score (median, IQR)			
Left hemisphere	2 (2)	1 (2)	2 (2)
Right hemisphere	2 (1)	2 (2)	2 (2)
Aβ + (***nn***, CSF Aβ_42_/ [^11^C] PiB-PET)	88/0	57/3	145/3
Phosphorylated tau (***n***, CSF p-tau +/-)	46/4	33/8	79/12
Total tau (***n***, CSF t-tau +/-)	44/6	33/8	77/14
Medication use (n/%)			
Anticholinergics	4 (5%)	6 (10%)	10 (7%)
Antidepressants	9 (10%)	5 (8%)	14 (9%)
AEDs	1 (1%)	3 (5%)	4 (3%)
Antipsychotics	1 (1%)	1 (2%)	2 (1%)
Benzodiazepines	3 (3%)	5 (8%)	8 (5%)

*SD* Standard deviation, *MMSE* Mini mental state examination, *IQR* Inter-quartile range, *MTA* Medial temporal lobe atrophy, *Aβ* + Amyloid beta positive, as measured by cerebrospinal fluid Aβ42 concentration or [15] C-labeled Pittsburgh Compound-B positron emission topography examination, *AEDs* Anti-epileptic drugs

^**^p < .01, *** *p* < .001

To analyze the differences in development over time between subgroups, we stratified subjects based on *baseline* disease stage (i.e., MCI, AD dementia). To mimic real-life conditions in the context of clinical trial design, we modeled the course of EEG measures without including knowledge on future clinical progression. We modeled and compared the development of the EEG outcome measures over time between groups by adding *Time*, *Group*, and Time*Group interaction variables to the model. Again, a separate LMM was built for each measure of interest. To investigate whether sex, age, or medication use influenced the (difference between the) development of the groups over time, we performed both crude and covariate adjusted analysis. We report regression coefficients with 95% confidence intervals. A *p*-value < 0.05 was considered statistically significant. Due to the exploratory nature of the study, adjustment for multiple comparisons was not performed. Details of the LMM analyses are included in Additional file 1.

#### Effect- and sample size calculation

Effect sizes (measured as *d*) of the strongest whole-group and group-wise LMM results were computed *per month* using the formula below [57]. Yearly and two-yearly effect sizes were linearly estimated.

$$d= \frac{\mathrm{Difference\,between\,the\,means}\,(\beta )}{\sqrt{{\mathrm{var}}_{\mathrm{intercept}}+{\mathrm{var}}_{\mathrm{slope}*}+ {\mathrm{var}}_{\mathrm{residual}}}}$$

*Var_slope_ was set to zero for models that only included a random intercept on subject level.

In the context of a clinical trial, different treatment effects on EEG outcome measures are imaginable. In a stabilizing scenario (1), a treatment effectively halts the progression of EEG abnormalities over time. Treatment effectiveness in this scenario corresponds to *d*, as computed from the LMM results. A treatment can also slow down the rate of deterioration of EEG outcome measures, which we will refer to as a “less deterioration” scenario (2). This second scenario results in a *smaller* treatment effect size than the stabilizing scenario. Finally, deterioration of EEG outcome measures (e.g., oscillatory slowing) may be reversed by a treatment. Improvement of the EEG measures in the active group results in a *larger* treatment effect size than would be observed in a stabilizing scenario (3).

We estimated minimum sample sizes required to detect different treatment effects (i.e., 50% less deterioration, stabilization, or 50% improvement) on EEG outcome measures in hypothetical clinical trials of 1- or 2-year duration, with EEG measurements taken at baseline and end-of-treatment. This was done using G*Power software [58] at 0.05 to 99% power, for a one-sided two-sample *t*-test, with a type I error (*α*) of *p* < 0.05. A balanced design was assumed.

## Results

### Baseline characteristics

Eighty-eight amyloid-positive MCI and 60 AD dementia subjects were included in this study. Table 2 summarizes the baseline demographic and clinical characteristics of the study population. The mean age of subjects with MCI was 70.7 years (SD 7.2). This was significantly higher than the mean age of AD dementia subjects (66.9 years, SD 8.2) (*p* < 0.01). We report significantly higher median MMSE scores (available for 143 subjects at baseline) for the MCI (27, IQR 3) than the AD dementia group (24, IQR 5) (*p* < 0.001). For a more comprehensive overview of neuropsychological test scores of the participants at baseline, see Table S1 & S2 in Additional file 2. Sex ratio and MTA scores (*n* = 84) did not differ significantly between groups at baseline. CSF p-tau and t-tau levels were available for 91 patients.

**Fig. 1:**
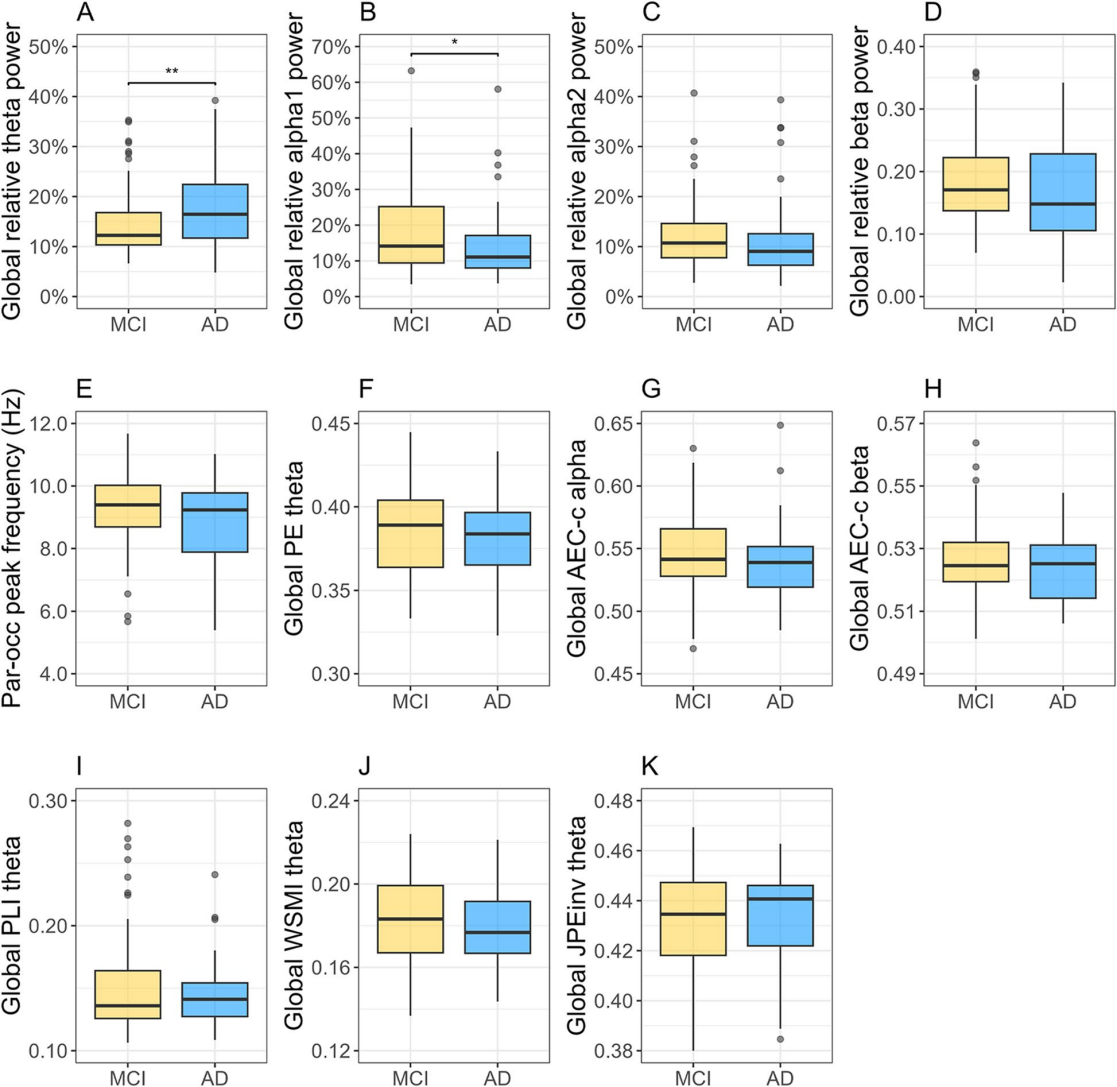
**A**-**K** Baseline comparisons of global EEG measures and the parieto-occipital peak frequency between Aβ + MCI and AD dementia patients. **A**-**D** Global relative *theta, alpha1, alpha2*, and *beta* power. **E** Parieto-occipital peak frequency in Hz. **F** Global permutation entropy (PE) *theta*. **G**-**H** Corrected amplitude envelope correlation (AEC-c) *alpha* and *beta*. **I** Phase lag index (PLI) *theta*. **J** Weighted symbolic mutual information (wSMI) *theta*. **K** Inverted joint permutation entropy (JPE_inv_) *theta*. * *p* < .05, **p < .01

Baseline comparisons of global EEG measures and the parieto-occipital peak frequency between the MCI and AD dementia subgroups are displayed in Fig. 1. The MCI group had significantly lower global relative *theta* power (*p* < 0.01) and higher relative *alpha1* power values (*p* < 0.05) compared to the AD dementia group (Fig. 1A, B). The groups did not differ significantly with respect to the remaining measures. Mean (SD) values for all measures are reported in Additional file 2 (Table S3).

### Follow-up characteristics

Participants underwent 2 to 4 EEG recordings within a follow-up period of 2.6 to 35.5 months (Table 3). Reasons for follow-up included clinical (re-)evaluation, screening for potential inclusion in a clinical trial, or participation in a clinical trial (as control subject). The MCI group had a longer median follow-up time (11.0 months (range 2.6 – 34.8)) than the AD dementia group (5.9 months (range 2.8 – 35.5)). This difference was however not statistically significant.

**Table 3:** Follow-up characteristics

	**MCI**	**AD dementia**	**Total**
Reason for follow-up (*n*)			
Clinical follow-up	46	18	64
Clinical trial – screening phase	5	24	29
Clinical trial – control condition	37	18	55
Number of EEG recordings (median, range)	2 (2–4)	2 (2–3)	2 (2–4)
Total follow-up time, months (median, range)	11.0 (2.6 – 34.8)	5.9 (2.8 – 35.5)	7.2 (2.6 – 35.5)

### EEG measures’ development over time

#### Whole-group LMM

**Fig. 2:**
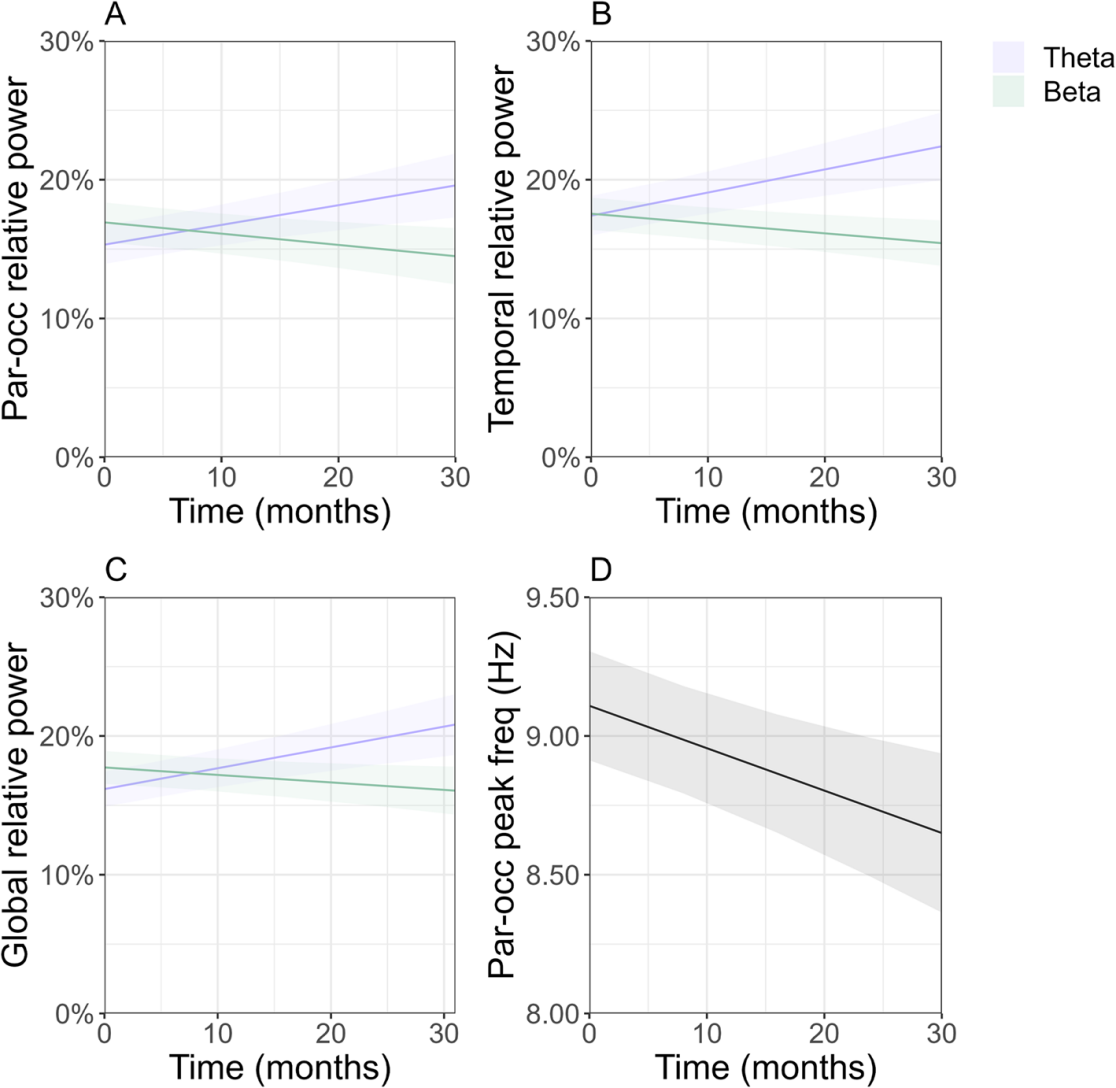
**A**-**D** Estimated trajectories and 95% confidence intervals for EEG measures in Aβ + AD patients (MCI and AD dementia). Significant whole-group estimates are visualized (*p* < .05). **A**-**C** Parieto-occipital, temporal, and global relative *theta* and *beta* power. **D** Parieto-occipital peak frequency in Hz

The whole-group LMM analysis revealed significant regional and global oscillatory slowing over time in our Aβ + AD cohort (*N* = 148), with strongest effects for regional measures. We found a significant increase in parieto-occipital, temporal, and global relative *theta* power, as well as a significant decrease in parieto-occipital, temporal and global relative *beta* power (Fig. 2A-C) and parieto-occipital peak frequency (Fig. 2D) over time. Although we report no significant longitudinal changes in functional connectivity strength and signal variability on whole-group level, estimates of regression coefficient β indicate a downward trend in parieto-occipital, temporal, and global AEC-c *alpha* and *beta* and JPE_inv_ *theta* connectivity, as well as PE *theta*. PLI *theta* and wSMI *theta* connectivity exhibit a consistent positive trend across all regions. Regression coefficients and 95% confidence intervals are reported in Table 4. Regression coefficient β reflects the change in outcome measure *per month*. For visualization purposes, only significant estimates are plotted in Fig. 2.

**Table 4:** Results of the whole-group LMM analysis performed to evaluate change in EEG measures over time (*in months*) in Aβ + AD patients (MCI and AD dementia). Regression coefficients (β) and 95% confidence intervals (CI) are reported

Region	Measure	Frequency band	β	95% CI		*p*-value
Parieto-occipital	Relative power	Theta^b^	1.4E-03	7.3E-04	2.2E-03	< .001***
		Alpha1^a^	-2.9E-04	-1.4E-03	8.5E-04	0.61
		Alpha2^a^	-2.6E-04	-1.3E-03	7.8E-04	0.62
		Beta^a^	-8.1E-04	-1.4E-03	-2.3E-04	< .01**
	Peak frequency	-^a^	-1.5E-02	-2.4E-02	-6.8E-03	< .001**
	AEC-c	Alpha^a^	-7.5E-05	-4.2E-04	2.7E-04	0.67
		Beta^a^	-9.3E-05	-2.2E-04	3.7E-05	0.16
	PLI	Theta^a^	2.0E-04	-2.0E-04	6.0E-04	0.33
	PE	Theta^a^	-9.7E-05	-2.7E-04	7.4E-05	0.27
	wSMI	Theta^a^	5.0E-05	-7.8E-05	1.8E-04	0.44
	JPE_inv_	Theta^a^	-5.3E-05	-1.6E-04	5.3E-05	0.32
Temporal	Relative power	Theta^a^	1.7E-03	9.2E-04	2.4E-03	< .001***
		Alpha1^a^	-4.5E-04	-1.1E-03	2.5E-04	0.21
		Alpha2^a^	-3.8E-04	-8.3E-04	6.5E-05	0.09
		Beta^a^	-7.1E-04	-1.2E-03	-2.4E-04	< .01**
	AEC-c	Alpha^a^	-9.8E-05	-4.2E-04	2.2E-04	0.55
		Beta^a^	-3.6E-05	-1.6E-04	9.0E-05	0.57
	PLI	Theta^a^	1.3E-04	-2.3E-04	4.9E-04	0.49
	PE	Theta^a^	-1.6E-04	-3.2E-04	-3.6E-08	0.05
	wSMI	Theta^a^	7.4E-05	-4.5E-05	1.9E-04	0.22
	JPE_inv_	Theta^a^	-8.4E-05	-1.7E-04	5.3E-06	0.07
Global	Relative power	Theta^b^	1.5E-03	8.6E-04	2.2E-03	< .001***
		Alpha1^a^	-4.6E-04	-1.3E-02	3.4E-04	0.26
		Alpha2^a^	-3.1E-04	-9.2E-04	3.0E-04	0.32
		Beta^a^	-5.4E-04	-1.0E-03	-4.6E-05	< .05*
	AEC-c	Alpha^a^	-9.2E-05	-4.1E-04	2.3E-04	0.57
		Beta^a^	-5.8E-05	-1.8E-04	6.2E-05	0.34
	PLI	Theta^a^	1.5E-04	-1.9E-04	5.0E-04	0.38
	PE	Theta^a^	-1.3E-04	-2.8E-04	2.8E-05	0.11
	wSMI	Theta^a^	6.5E-05	-5.4E-05	1.8E-04	0.28
	JPE_inv_	Theta^a^	-5.5E-05	-1.6E-04	4.7E-05	0.29

*AEC-c* Corrected amplitude envelope correlation, *PLI* Phase lag index, *PE* Permutation entropy, *wSMI* Weighted symbolic mutual information, *JPE*_*inv*_ Inverted joint permutation entropy

*p<.05, **p<.01, ***p<.001

^a^Random intercept on subject level

^b^Random intercept and random slope on subject level

#### Group-wise LMM

Group-wise LMM analysis was performed to investigate potential differences in the development of EEG measures over time between patients in different stages of AD. Tables 5, 6 and 7 present the regression coefficients and 95% confidence intervals of the *Time* effects for each group (i.e., MCI and AD dementia). None of the EEG measures showed significant Time*Group interactions. Note that regression coefficient β of the interaction effects indicates the difference in *Time* effect between the MCI and AD dementia subgroups. We report a negligible effect on the rates of change of the investigated EEG measures by the addition of sex, age, and medication use covariates to the LMMs (see Additional file 2, Table S4-6), and therefore make use of the uncorrected results in the remainder of the paper.

**Table 5:** Results of the group-wise LMM analysis performed to evaluate change in parieto-occipital EEG measures over time (*in months*) in Aβ + MCI or AD dementia patients. Regression coefficients (β) and 95% confidence intervals (CI) of the *Time* effects are reported. Estimates for the Group*Time interaction effects, as well as estimates adjusted for age, sex and medication use are presented in Additional file 2 (Table S4)

Region	Measure	Frequency band	Disease stage	*β*	95% CI		*p*-value
Parieto-occipital	Relative power	Theta	MCI^b^	1.6E-03	8.3E-04	2.3E-03	< .001***
			AD dementia^b^	8.7E-04	-2.0E-04	1.9E-03	0.12
		Alpha1	MCI^a^	3.8E-04	-1.1E-03	1.9E-03	0.62
			AD dementia^a^	-1.2E-03	-3.0E-03	5.2E-04	0.17
		Alpha2	MCI^a^	-1.3E-04	-1.5E-03	1.2E-03	0.86
			AD dementia^a^	-4.5E-04	-2.0E-03	1.1E-03	0.58
		Beta	MCI^a^	-1.0E-03	-1.8E-03	-2.7E-04	< .01**
			AD dementia^a^	-5.0E-04	-1.4E-03	3.9E-04	0.27
	Peak frequency	-	MCI^a^	-1.0E-02	-2.1E-02	1.2E-03	0.08
			AD dementia^a^	-2.3E-02	-3.6E-02	-9.6E-03	< .001***
	AEC-c	Alpha	MCI^a^	-1.5E-04	-6.1E-04	3.1E-04	0.53
			AD dementia^a^	2.1E-05	-5.1E-04	5.5E-04	0.94
		Beta	MCI^a^	-7.1E-05	-2.4E-04	9.9E-05	0.41
			AD dementia^a^	-1.2E-04	-3.2E-04	7.5E-05	0.22
	PLI	Theta	MCI^a^	1.9E-04	-3.4E-04	7.2E-04	0.48
			AD dementia^a^	2.1E-04	-4.1E-04	8.3E-04	0.50
	PE	Theta	MCI^a^	-2.4E-05	-2.5E-04	2.0E-04	0.84
			AD dementia^a^	-2.0E-04	-4.6E-04	6.4E-05	0.14
	wSMI	Theta	MCI^a^	-1.8E-05	-1.9E-04	1.5E-04	0.83
			AD dementia^a^	1.4E-04	-5.2E-05	3.4E-04	0.15
	JPE_inv_	Theta	MCI^a^	7.6E-07	-1.4E-04	1.4E-04	0.99
			AD dementia^a^	-1.3E-04	-2.9E-04	3.6E-05	0.13

*AEC-c* Corrected amplitude envelope correlation, *PLI* Phase lag index, *PE* Permutation entropy, *wSMI* Weighted symbolic mutual information, *JPE*_*inv*_ Inverted joint permutation entropy

*p<.05, **p<.01, ***p<.001

^a^Random intercept on subject level

^b^Random intercept and random slope on subject level

**Table 6:** Results of the group-wise LMM analysis performed to evaluate change in temporal EEG measures over time (*in months*) in Aβ + MCI or AD dementia patients. Regression coefficients (β) and 95% confidence intervals (CI) of the *Time* effects are reported. Estimates for the Group*Time interaction effects, as well as estimates adjusted for age, sex, and medication use are presented in Additional file 2 (Table S5)

Region	Measure	Frequency band	Disease stage	β	95% CI		*p*-value
Temporal	Relative power	Theta	MCI^b^	2.2E-03	1.2E-03	3.1E-03	< .001***
			AD dementia^b^	8.2E-04	-3.1E-04	2.0E-03	0.16
		Alpha1	MCI^b^	-3.7E-04	-1.3E-03	5.6E-04	0.44
			AD dementia^b^	-6.0E-04	-1.7E-03	5.0E-04	0.30
		Alpha2	MCI^a^	-2.5E-04	-8.4E-04	3.4E-04	0.41
			AD dementia^a^	-5.7E-04	-1.3E-03	1.1E-04	0.10
		Beta	MCI^a^	-8.0E-04	-1.4E-03	-1.8E-04	< .05*
			AD dementia^a^	-5.8E-04	-1.3E-03	1.5E-04	0.12
	AEC-c	Alpha	MCI^a^	-1.6E-04	-5.8E-04	2.7E-04	0.47
			AD dementia^a^	-2.2E-05	-5.1E-04	4.7E-04	0.93
		Beta	MCI^a^	-2.5E-05	-1.9E-04	1.4E-04	0.77
			AD dementia^a^	-5.2E-05	-2.5E-04	1.4E-04	0.60
	PLI	Theta	MCI^a^	1.1E-04	-3.7E-04	5.8E-04	0.66
			AD dementia^a^	1.5E-04	-4.0E-04	7.1E-04	0.59
	PE	Theta	MCI^a^	-9.2E-05	-3.0E-04	1.2E-04	0.39
			AD dementia^a^	-2.5E-04	-4.9E-04	-7.6E-06	< .05*
	wSMI	Theta	MCI^a^	1.5E-05	-1.4E-04	1.7E-04	0.85
			AD dementia^a^	1.5E-04	-2.8E-05	3.4E-04	0.10
	JPE_inv_	Theta	MCI^a^	-5.0E-05	-1.7E-04	6.7E-05	0.40
			AD dementia^a^	-1.3E-04	-2.7E-04	6.9E-06	0.06

*AEC-c* Corrected amplitude envelope correlation, *PLI* Phase lag index, *PE* Permutation entropy, *wSMI* Weighted symbolic mutual information, *JPE*_*inv*_ Inverted joint permutation entropy

*p<.05, **p<.01, ***p<.001

^a^Random intercept on subject level

^b^Random intercept and random slope on subject level

**Table 7:** Results of the group-wise LMM analysis performed to evaluate change in global EEG measures over time (*in months*) in Aβ + MCI or AD dementia patients. Regression coefficients (*β*) and 95% confidence intervals (CI) of the *Time* effects are reported. Estimates for the Group*Time interaction effects, as well as estimates adjusted for age, sex and medication use are presented in Additional file 2 (Table S6)

Region	Measure	Frequency band	Disease stage	*β*	95% CI		*p*-value
Global	Relative power	Theta	MCI^b^	1.8E-03	1.1E-03	2.6E-03	< .001***
			AD dementia^b^	9.3E-04	-4.6E-05	1.9E-03	0.07
		Alpha1	MCI^b^	-2.4E-04	-1.3E-03	7.8E-04	0.64
			AD dementia^b^	-9.9E-04	-2.2E-03	1.8E-04	0.10
		Alpha2	MCI^a^	-1.9E-04	1.0E-03	6.2E-04	0.64
			AD dementia^a^	-4.8E-04	-1.4E-03	4.6E-04	0.32
		Beta	MCI^a^	-7.0E-04	-1.3E-03	-5.1E-05	< .05*
			AD dementia^a^	-3.2E-04	-1.1E-03	4.3E-04	0.40
	AEC-c	Alpha	MCI^a^	-1.7E-04	-5.9E-04	2.5E-04	0.42
			AD dementia^a^	1.3E-05	-4.7E-04	5.0E-04	0.96
		Beta	MCI^a^	-3.4E-05	-1.9E-04	1.2E-04	0.67
			AD dementia^a^	-9.1E-05	-2.7E-04	9.3E-05	0.33
	PLI	Theta	MCI^a^	1.3E-04	-3.2E-04	5.8E-04	0.58
			AD dementia^a^	1.8E-04	-3.5E-04	7.0E-04	0.50
	PE	Theta	MCI^a^	-5.5E-05	-2.6E-04	1.5E-04	0.60
			AD dementia^a^	-2.3E-04	-4.6E-04	1.2E-05	0.06
	wSMI	Theta	MCI^a^	2.9E-06	-1.5E-04	1.6E-04	0.97
			AD dementia^a^	1.5E-04	-3.3E-05	3.3E-04	0.11
	JPE_inv_	Theta	MCI^a^	-5.6E-06	-1.4E-04	1.3E-04	0.93
			AD dementia^a^	-1.2E-04	-2.8E-04	3.4E-05	0.12

*AEC-c* Corrected amplitude envelope correlation, *PLI* Phase lag index, *PE* Permutation entropy, *wSMI* Weighted symbolic mutual information, *JPE*_*inv*_ Inverted joint permutation entropy

*p<.05, **p<.01, ***p<.001

^a^Random intercept on subject level

^b^Random intercept and random slope on subject level

**Fig. 3:**
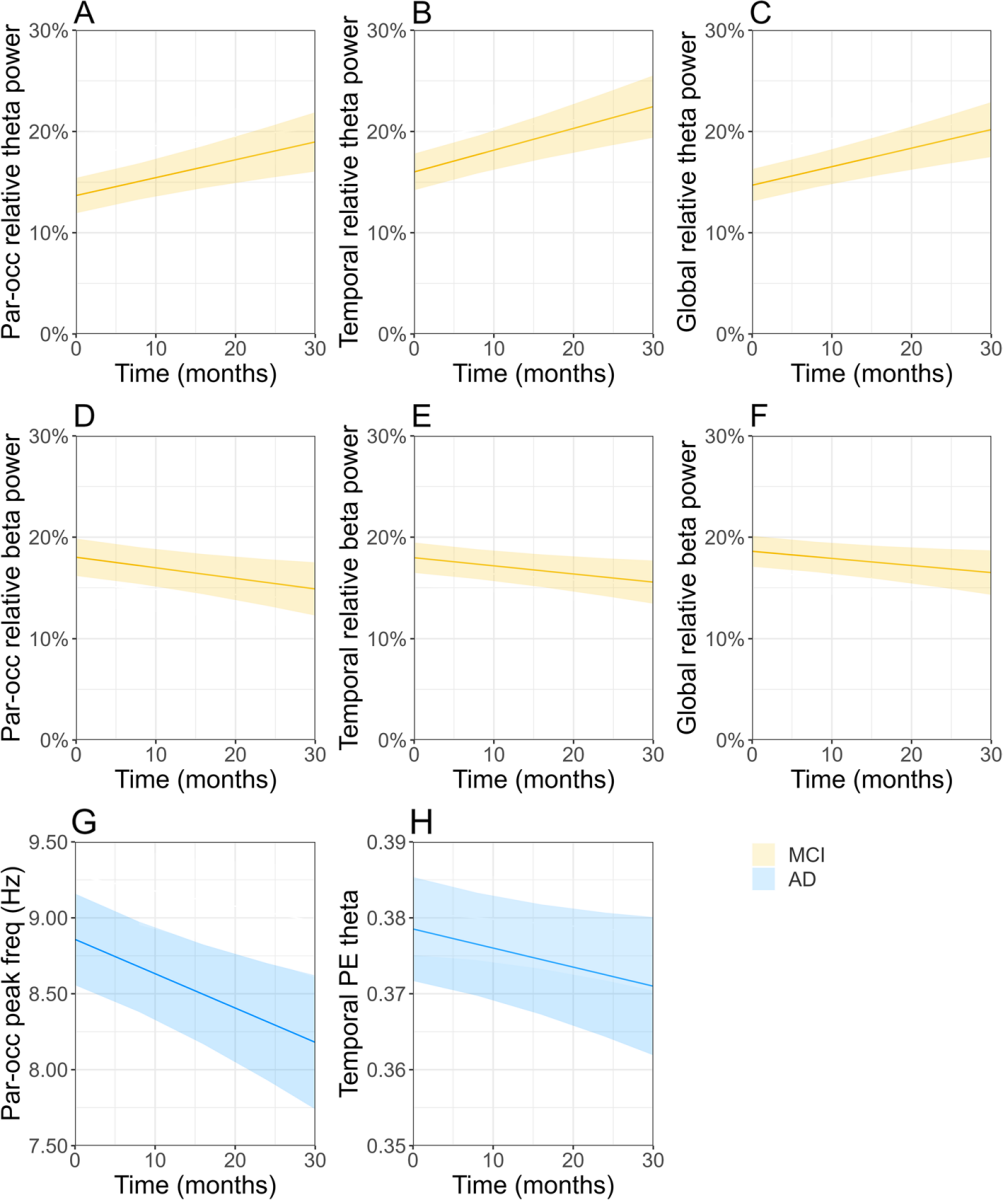
**A**-**F** Estimated trajectories and 95% confidence intervals for EEG measures in Aβ + MCI or AD dementia patients. Significant group-wise estimates are visualized (*p* < .05). **A**-**C** Parieto-occipital, temporal, and global relative *theta* power, MCI (*yellow*). **D**-**F** Parieto-occipital, temporal, and global relative relative *beta* power, MCI. **G** Parieto-occipital peak frequency (in Hz), AD dementia *(blue)*. **H** Temporal permutation entropy (PE) *theta*, AD dementia

Electrophysiological deterioration was most prominent in MCI subjects, reflected by significant development of 6 EEG measures over time (Fig. 3A-F). Similar to the whole-group analysis, strongest effects were reported for regional measures (Tables 5 and 6). We found a significant increase in parieto-occipital, temporal, and global relative theta power (Fig. 3A-C), while a significant decrease was demonstrated for parieto-occipital, temporal, and global relative *beta* power (Fig. 3D-F). The AD dementia group showed a significant decrease of the parieto-occipital peak frequency and temporal PE *theta* over time. The direction of reported effects for the remaining measures was largely similar across groups (Tables 5, 6 and 7). We report a downward trend (although not significant) in parieto-occipital, temporal, and global AEC-c *beta*, relative *alpha2* power and PE *theta*, as well as a positive trend in PLI *theta* connectivity. Global and temporal *alpha1* power and JPE_inv_ connectivity decreased in both groups, whereas wSMI *theta* connectivity increased over time.

#### EEG measures sensitive to change

Table 8 displays the subset of EEG measures that was included in the second part of the study. The EEG measures most sensitive to change in the whole-group LMM analysis included temporal relative *theta* and *beta* power and the parieto-occipital peak frequency. For the group-wise LMM analysis, this included temporal relative *theta* power and parieto-occipital relative *beta* power for MCI patients and the parieto-occipital peak frequency and temporal PE *theta* for AD dementia patients.

**Table 8:** Subset of EEG measures most sensitive to change

Analyzed subjects	Region	Measure	Frequency band
Whole-group	Temporal	Relative power	Theta, beta
	Parieto-occipital	Peak frequency	-
MCI	Temporal	Relative power	Theta
	Parieto-occipital	Relative power	Beta
AD dementia	Parieto-occipital	Peak frequency	-
	Temporal	Permutation entropy	Theta

#### Longitudinal effect sizes

To effectively compare the relative sensitivity to change of the EEG measures, yearly and two-yearly whole-group and group-wise effect sizes were computed from the LMM results. The largest longitudinal effect size was reported for temporal relative *theta* power (1 year: *d* = 0.23, 2 years: *d* = 0.45), followed by the parieto-occipital peak frequency (1 year: *d* = 0.15, 2 years: *d* = 0.30) and temporal relative *beta* power (1 year: *d* = 0.12, 2 years: *d* = 0.24). Larger effect sizes were reported for the individual MCI (1 year: temporal relative *theta* power *d* = 0.30, parieto-occipital *beta* power *d* = 0.14, 2 years: temporal relative *theta* power *d* = 0.60, parieto-occipital *beta* power *d* = 0.28) and AD dementia subgroups (1 year: parieto-occipital peak frequency *d* = 0.23, temporal PE *theta d* = 0.11, 2 years: parieto-occipital peak frequency *d* = 0.45, temporal PE *theta d* = 0.22). Effect sizes for different treatment scenarios (i.e., 50% less deterioration and 50% improvement) are displayed in Table S7 & S8 in Additional file 2.

#### Sample size calculations

**Fig. 4:**
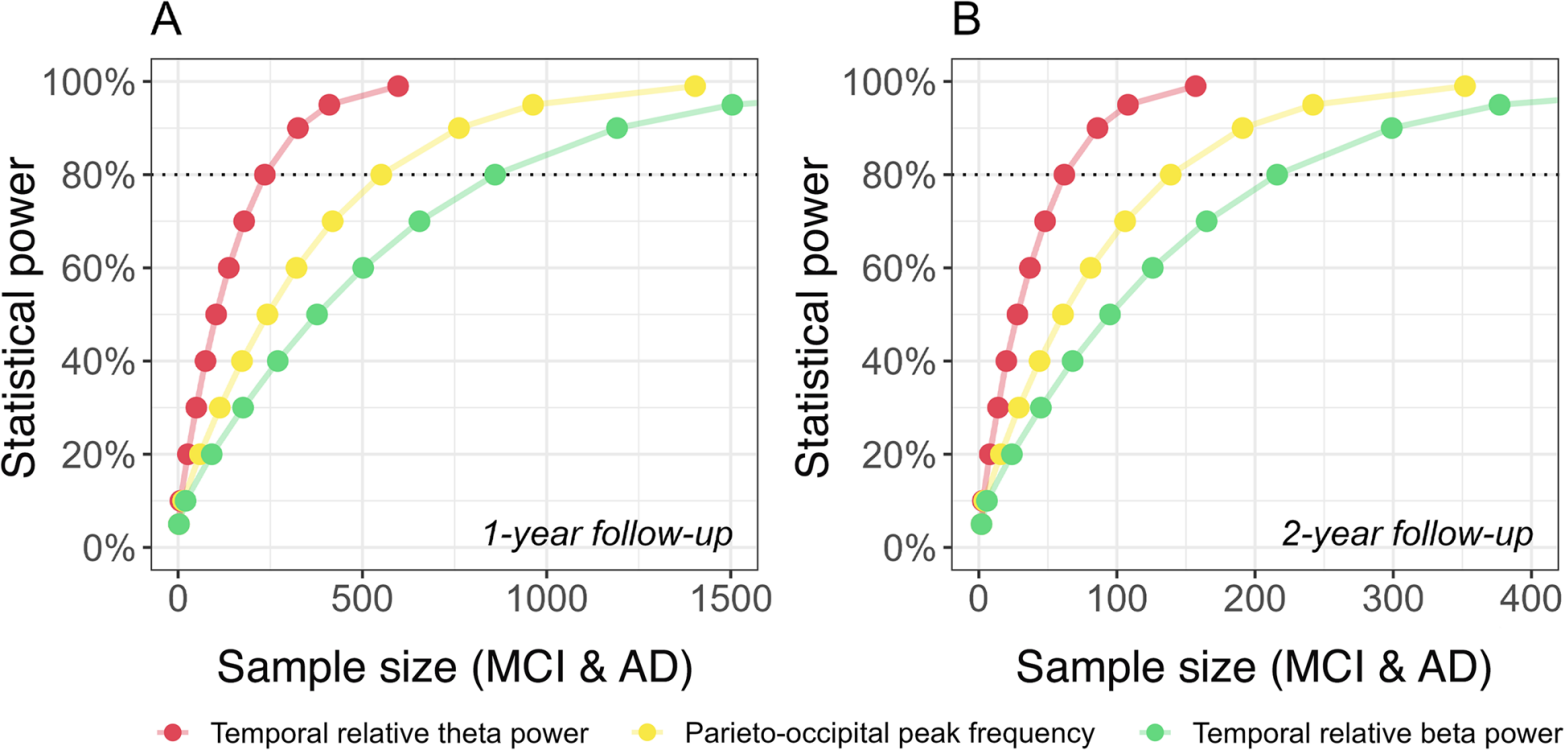
**A**-**B** Statistical power as a function of sample size (Aβ + MCI and AD dementia). The minimum required sample size per arm was estimated at 0.05 to 99% power for 1-year (**A**) and 2-year (**B**) trial duration. Effect sizes for EEG outcome measures were computed based on significant whole-group LMM results (Table S7, Additional file 2)

We estimated the number of participants required to demonstrate different treatment effects on the development of EEG outcome measures over time in hypothetical clinical trials of 1- or 2-year duration. For a 1-year trial, including both MCI and AD dementia patients, the minimum sample size *per arm* required to detect a stabilizing effect on progression of temporal relative *theta*, the parieto-occipital peak frequency, and temporal relative *beta* power at 80% power was 235, 551, and 860, respectively. For trials with a duration of 2 years, these estimates decreased by a factor of four to 62, 139, and 216 (Fig. 4, Table 9).

**Table 9:** Required sample sizes (per arm) to detect different treatment effects on EEG outcome measures in Aβ + AD patients (MCI and AD dementia) in a hypothetical trial of 1- or 2-year duration, at 80% power

Subjects	Region	Frequency band	Measure	Sample size (50% less deterioration)		Sample size (stabilization)		Sample size (50% improvement)	
				1 year	2 years	1 year	2 years	1 year	2 years
**Whole-group (MCI** and** AD dementia)**	Temporal	Theta	Relative power	1023	235	235	62	108	28
	Parieto-occipital	-	Peak frequency	2525	551	551	139	257	62
	Temporal	Beta	Relative power	3436	860	860	216	383	102

**Fig. 5:**
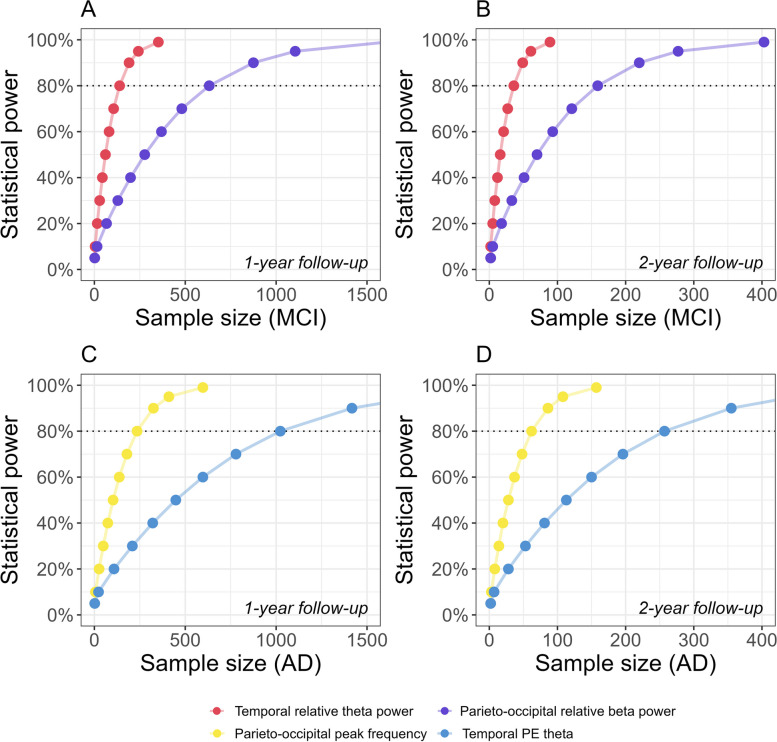
**A**-**D** Statistical power as a function of sample size (Aβ + MCI or AD dementia patients). The minimum required sample size per arm was estimated at 0.05 to 99% power for 1-year (**A**, **C**) and 2-year (**B**, **D**) trial duration. Effect sizes for EEG outcome measures were computed based on significant group-wise LMM results (Table S8, Additional file 2)

Stratification based on disease severity at baseline revealed differences in the development of EEG measures over time between MCI and AD dementia patients (Fig. 3, Tables 5, 6 and 7). Not all EEG measures showed significant change over time in each group. Figure 5 and Table 10 display sample size estimates for hypothetical trials that would specifically target MCI (Fig. 5A, B) or AD dementia patients (Fig. 5C, D). Minimum required sample sizes are substantially lower for trials focusing on a single disease stage. To reliably assess a stabilizing treatment effect in MCI patients in a 2-year trial, the minimum required sample size per arm would be 36 or 159 for temporal relative *theta* power and parieto-occipital relative *beta* power, respectively. The parieto-occipital peak frequency and temporal PE *theta* are most sensitive to change in AD dementia patients. Two-year AD dementia trials should include a minimum of 62 or 257 patients per arm to detect a stabilizing treatment effect on the respective measures. As expected, required sample sizes are substantially larger for treatments that slow down (rather than stabilize) the rate of deterioration of EEG, whereas required sample sizes are smaller for treatments that reverse deterioration (Tables 9 and 10).

**Table 10:** Required sample sizes (per arm) to detect different treatment effects on EEG outcome measures in Aβ + MCI or AD dementia patients, in a hypothetical trial of 1- or 2-year duration, at 80% power

Subjects	Region	Frequency band	Measure	Sample size (50% less deterioration)		Sample size (stabilization)		Sample size (50% improvement)	
				1 year	2 years	1 year	2 years	1 year	2 years
**MCI**	Temporal	Theta	Relative power	551	139	139	36	62	17
	Parieto-occipital	Beta	Relative power	2525	632	632	159	282	71
**AD dementia**	Parieto-occipital	-	Peak frequency	1023	235	235	62	108	28
	Temporal	Theta	PE	3436	1023	1023	257	429	115

## Discussion

This retrospective longitudinal EEG study demonstrates significant deterioration of global, parieto-occipital, and temporal resting-state EEG measures over time (including relative *theta*, *beta* power, *theta* band signal variability, and peak frequency) in Aβ + patients with MCI or dementia due to AD. Effects were measurable after a period as short as 1 month. We provide support for the inclusion of EEG outcome measures in AD clinical trials, as their fast rate of deterioration may facilitate early detection of treatment effects on neuronal function. EEG measures’ sensitivity to change depended on the region-of-interest and the disease severity of subjects. The effect of baseline age, gender, and medication use on the development of the EEG measures over time was deemed negligible based on covariate adjusted analysis. When designing a trial with 1-year follow-up, the estimated sample size per arm (with two arms and 1:1 randomization, at 80% power) required to detect a *stabilizing* treatment effect on temporal relative *theta* power and parieto-occipital relative *beta* power in MCI patients was 139 or 632, respectively. For a 2-year follow-up period, these numbers were reduced to 36 or 159 MCI patients per arm. When a treatment is expected to slow down (rather than *stabilize*) the deterioration of EEG measures, it is advisable to set up a more conservative trial with larger sample sizes. Conversely, if a treatment is hypothesized to reverse deterioration of EEG measures, smaller sample sizes can be considered.

Our results are in line with previous studies reporting spectral EEG measures as most consistent for monitoring AD progression [59] and response to interventions in AD clinical trials [60, 61]. In agreement with our hypothesis, most prominent effects were localized in temporal regions, with highest sensitivity for relative *theta* power. This is consistent with earlier findings [13, 14, 35, 62].

Disease severity at baseline influenced EEG measures’ rates of change, with fastest deterioration reported in MCI subjects. The MCI group showed significant development over time for 6 EEG measures, including parieto-occipital, temporal, and global relative *theta* and *beta* power. AD dementia patients showed a significant decrease of the parieto-occipital peak frequency and temporal PE *theta*. We do not report significant *Time x Group* effects (Additional file 2, Table S4-6), indicating that the development of EEG measures over time did not differ significantly between groups.

### Underlying mechanisms

A growing body of evidence suggests that large-scale circuit and network function are affected by a neuronal excitation/inhibition (E/I) imbalance in AD (for a recent review, see [63]). Neuronal hyperactivity has been demonstrated in early stages of AD, both in animal models (using in vivo calcium imaging, [64, 65] and human EEG data (using spike detection, [66]). There is substantial evidence that soluble Aβ is crucial for this increase in neuronal activity [67, 68]. Soluble tau has on the other hand been associated with the silencing of neuronal activity. The presence of neurofibrillary tangles has been linked to changes in the number and morphology of dendritic spines in pyramidal cells of AD patients. Considering that dendritic spines are fundamental structures in memory, learning, and cognition, this is thought to be a key event in AD pathogenesis [69]. Abnormal spectral power and functional connectivity within the *alpha* and *delta-theta* frequency ranges have previously been shown to be differentially associated with Aβ and tau accumulations in patients with AD [70, 71]. Microscale hyperactivity has moreover been linked to the large-scale oscillatory slowing of M/EEG signals that is observed in AD patients, using a whole-brain computational network model [72]. The increase of relative *theta* power and decrease of relative *beta* power that we report in this study may be indirect measures of (Aβ-mediated) hyperactivity of pyramidal cells and/or interneuron dysfunction. This raises the question whether measures that quantify the E/I ratio of neuronal oscillations more directly could be more sensitive to change than conventional spectral measures. The validity of available EEG E/I ratio measures (e.g., functional E/I balance [73], fitting oscillations and one over f (“FOOOF”) [74]) however remains to be evaluated in further studies. The density of Aβ deposits and neurofibrillary tangles is known to vary across cortical regions and disease stages [75–77]. Studies correlating EEG measures with PET maps of Aβ and tau accumulation may provide an explanation for the regional- and group-differences demonstrated in this study.

### Sample size considerations

On whole-group level, largest effect sizes were reported for temporal relative *theta* and *beta* power and the parieto-occipital peak frequency. Temporal relative *theta* and parieto-occipital relative *beta* power exhibited largest effect sizes in MCI patients. AD dementia subjects displayed largest effect sizes for the parieto-occipital peak frequency and temporal PE *theta*. Corresponding sample sizes estimates were substantially lower for hypothetical trials focusing on a single disease stage than for trials including MCI *and* AD dementia patients. Clinical trials of phases 2 and 3 typically include 100 to 300 and 300 to 3000 patients in each patient group [78]. Our results suggest that EEG outcome measures are appropriate for trials of this size, particularly when outcome measures are tailored to the patient group under investigation (MCI or AD dementia). If these findings are replicated, they might even lower the minimum number of required trial participants. We did not differentiate between converters and non-converters in the MCI group. During the design stage of a clinical trial, information on future clinical progression of its participants is unknown. We therefore provided sample size estimates for groups categorized based on baseline diagnosis only.

### The role of EEG outcome measures in AD clinical trials

Finding tools with sufficient sensitivity to detect drug-placebo differences in pre-dementia stages of AD is challenging. EEG measures however appear to be sensitive to change in early stages of disease. They could play an important role in early stages of drug development, for instance by demonstrating target engagement. In drug development programs, a proof-of-concept (POC) Phase 2a study is typically performed to help a drug developer make a “Go/NoGo” decision based on the efficacy performance of a medical agent. POC can be based on a clinical response, a biomarker response or a combination of both types of outcome measures. So far, no biomarker has been granted surrogate status in AD drug development [6]. This means that proof of target engagement (i.e., biomarker changes induced by a therapy) does not guarantee clinical benefit at a later stage. Nevertheless, biomarker outcomes are important to understand the biological impact of an agent. EEG outcome measures could provide insight into (rescued or restored) circuit-level function. Moreover, a candidate treatment and its Phase 3 program can be de-risked by acquiring a set of biomarkers and clinical measures that support its potential effectiveness. Target engagement markers that are currently employed in AD clinical trials (i.e., fluid, imaging) are obtained using invasive and/or expensive techniques. EEG outcome measures could be a cost-effective, non-invasive alternative to demonstrate target engagement.

## Strengths

This study presents one of the largest and most comprehensive longitudinal EEG analyses in amyloid-positive AD patients to date. We provide recommendations for the design of future AD trials in which EEG measures will be used as secondary endpoints. In contrast to earlier longitudinal EEG studies [32, 33, 35–38], diagnoses were based on recent diagnostic guidelines and extensive diagnostic screening. We employed linear mixed effects models to model the development of a large variety of global and regional EEG measures over time. LMMs are known for their ability to handle missing data points and variable measurement schedules between individuals and are therefore highly valuable when analyzing a clinical dataset [79]. Our findings also emerged after controlling for baseline age, sex, and medication use, adding to their validity.

## Limitations

As discussed in the "Underlying mechanisms" section, the presence of AD pathology is associated with changes in neuronal activity, which consequently affect EEG measures. All AD patients included in this study were positive for amyloid deposition. The extent of tau pathology and neurodegeneration (as assessed using cerebrospinal fluid p-tau and t-tau levels) however varied between patients (see Table 1) and was not characterized for the full cohort. This likely contributed to increased variability in the development of EEG measures over time among patients. Our results are not consistent with studies reporting changes in functional connectivity strength over time in AD patients [15, 32]. This includes a decrease in *alpha* and *beta* band AEC-c in temporal and parietal regions [80] and increased *theta* band PLI [81]. We also do not report significant differences in global *alpha 2* and *beta* power, PE *theta*, AEC-c *alpha* and *beta,* PLI *theta,* wSMI *theta* and JPE_inv_ *theta* between MCI and AD dementia patients at baseline, as was previously reported in cross-sectional studies [14, 15, 54, 55]. These discrepancies may be explained by a potential selection bias in the clinical part of our dataset. While EEG recording is part of the standardized diagnostic work-up of our center [41], it is not a routine examination during follow-up visits. Clinicians may request an additional EEG recording in situations where there is uncertainty about a patients’ diagnosis, or when aberrant symptoms or progression profiles are observed. As a result, our clinical cohort may be somewhat atypical compared to the general Alzheimer population, also reflected by the high age of MCI compared to AD dementia subjects at baseline. When LMM analysis was performed for the subset of patients that participated in a clinical trial (*n* = 55) (see Additional file 2, Table S9), reported effect sizes were considerably larger. This suggests that the well-controlled patient inclusion process of clinical trials may further reduce the minimum sample size required to detect a treatment effect on EEG outcome measures. In the future, we aim to (re)perform longitudinal EEG analysis within the context of AD clinical trials. Including a larger number of subjects with structured follow-up visits will improve longitudinal estimates and will allow for a better comparison of the development of EEG measures between patients in different disease stages. Analyzing data from more than two time-points for each subject would also allow potential non-linear aspects of change to be captured. Moreover, we wish to evaluate the potential value of combined (e.g., Hub Disruption Index [82], Pathological Oscillatory Slowing Index [83]), and less conventional EEG outcome measures for longitudinal effect monitoring in AD clinical trials.

## Conclusion

The presented findings indicate that EEG measures, particularly spectral power, are promising secondary endpoints for AD interventions. They are sensitive to change over a short time period and have complementary value to neuroimaging biomarkers, by providing a more direct measurement of neurotransmission and synaptic activity. The selection of EEG outcome measures should be guided by the targeted disease stage (i.e., MCI or AD dementia). Based on our sample size estimations, EEG measures have potential to reduce the size, duration, and therefore costs of clinical trials, particularly for those aimed at slowing disease progression in MCI due to AD. Our study contributes to improved trial design, by enabling informed decision-making regarding the appropriate sample size for interpreting EEG results. Additional longitudinal studies are needed to validate these findings and to relate EEG measures more extensively to clinical and cognitive outcomes, ideally within the context of a clinical trial.

### Supplementary Information

**Additional file 1.** Supplementary methods. Details of the linear mixed effect model analyses.

**Additional file 2: Table S1.** Neuropsychological test results for memory clinic patients at baseline. **Table S2.** Neuropsychological test results for trial participants at baseline. **Table S3.** Comparison of baseline EEG measures between diagnostic groups. **Table S4.** Results of the group-wise LMM analysis performed to evaluate change in parieto-occipital EEG measures over time (in months) in Aβ+ MCI or AD dementia patients. **Table S5.** Results of the group-wise LMM analysis performed to evaluate change in temporal EEG measures over time (in months) in Aβ+ MCI or AD dementia patients. **Table S6.** Results of the group-wise LMM analysis performed to evaluate change in global EEG measures over time (in months) in Aβ+ MCI or AD dementia patients. **Table S7.** Longitudinal effect sizes of whole-group LMM results. **Table S8.** Longitudinal effect sizes of group-wise LMM results. **Table S9.** Three-month effect sizes (measured as Cohen’s *d*) for EEG measures that demonstrated a significant change over time in the trial-, clinical- and total dataset [84].

## Data Availability

The data which support the findings of this study are not freely available, but may be provided upon reasonable request. Due to the clinical nature of the data, a formal data sharing agreement is needed before any data can be shared.

## Abbreviations

Aβ( +): Amyloid beta (positive)
AD: Alzheimer’s disease
AEC-c: Corrected amplitude envelope correlation
AED: Antiepileptic drugs
CSF: Cerebrospinal fluid
EEG: Electroencephalography
JPE_inv_: Inverted joint permutation entropy
LMM: Linear mixed model
MCI: Mild cognitive impairment
MMSE: Mini mental state examination
MRI: Magnetic resonance imaging
MTA: Medial temporal lobe atrophy
PE: Permutation entropy
PET: Positron emission tomography
PLI: Phase lag index
POC: Proof of concept
SCD: Subjective cognitive decline
wSMI: Weighted symbolic mutual information
