# Supplementary material for: Longitudinal resting-state EEG in amyloid-positive patients along the Alzheimer’s disease continuum: considerations for clinical trials
Source: Alzheimers Res Ther. 2023 Oct 19;15:182. doi: 10.1186/s13195-023-01327-1 (PMC10585755; doi:10.1186/s13195-023-01327-1)
Supplement: Supplementary file 1 — Additional file 1. Supplementary methods. Details of the linear mixed effect model analyses. [file 13195_2023_1327_MOESM1_ESM.pdf]

Supplementary methods

Details of the linear mixed effect model analyses.

Main predictor: Time

The rate of change for each EEG measure was calculated as the individual slope per participant in a linear regression of measure by time. Linear mixed effects models (LMMs) were performed separately and independently for each outcome measure. We explain the univariate LMMs with example pseudocode for R (version 4.0.3, lme4 package).

The **whole-group LMMs** included one fixed effect (time from baseline) and one or two random effects:

1. The intercept per participant (capturing the fact that some participants have lower/higher baseline EEG values than others).
2. The slope per participant (capturing the possibility that the effect of time is not the same for all participants).

Model A1: Whole-group LMM (random intercept)

```
lmer(EEG_measure ~ time_from_baseline_mnths + (1|Individual_ID))
```

Model A2: Whole-group LMM (random intercept + random slope)

```
lmer(EEG_measure ~ time_from_baseline_mnths +
(1+ time_from_baseline_mnths |Individual_ID)))
```

The **group-wise LMM models** included the fixed effects time from baseline, baseline diagnosis and the interaction term time*baseline diagnosis. The models included a random intercept per participant. The interaction term time*baseline diagnosis was interpreted as the effect of the baseline diagnosis on the subsequent rate of biomarker change. The models were also evaluated by adjusting for age, sex and medication use.

Model B: Group-wise LMM (crude)

```
lmer(EEG_measure ~ time_from_baseline_mnths + diagnostic_group +
time_from_baseline_mnths: diagnostic_group + (1| Individual_ID))
```

Model C: Group-wise LMM (adjusted)

```
lmer(EEG_measure ~ time_from_baseline_mnths + diagnostic_group +
time_from_baseline_mnths: diagnostic_group + (1| Individual_ID))
+ Medication_score + Age + Sex)
```
