# Supplementary material for: Longitudinal resting-state EEG in amyloid-positive patients along the Alzheimer’s disease continuum: considerations for clinical trials
Source: Alzheimers Res Ther. 2023 Oct 19;15:182. doi: 10.1186/s13195-023-01327-1 (PMC10585755; doi:10.1186/s13195-023-01327-1)
Supplement: Supplementary file 2 — Additional file 2: Table S1. Neuropsychological test results for memory clinic patients at baseline. Table S2. Neuropsychological test results for trial participants at baseline. Table S3. Comparison of baseline EEG measures between diagnostic groups. Table S4. Results of the group-wise LMM analysis performed to evaluate change in parieto-occipital EEG measures over time (in months) in Aβ+ MCI or AD dementia patients. Table S5. Results of the group-wise LMM analysis performed to evaluate change in temporal EEG measures over time (in months) in Aβ+ MCI or AD dementia patients. Table S6. Results of the group-wise LMM analysis performed to evaluate change in global EEG measures over time (in months) in Aβ+ MCI or AD dementia patients. Table S7. Longitudinal effect sizes of whole-group LMM results. Table S8. Longitudinal effect sizes of group-wise LMM results. Table S9. Three-month effect sizes (measured as Cohen’s d) for EEG measures that demonstrated a significant change over time in the trial-, clinical- and total dataset [84]. [file 13195_2023_1327_MOESM2_ESM.pdf]

Supplementary results

Table S1. Neuropsychological test results of memory clinic patients at baseline (n=93). Unstandardized scores are presented (median with interquartile range).

Neuropsychological assessment	Primary outcome measure	MCI	n	AD	n	Total	n
MMSE	Total score (out of 30)	27 (2)	51	23 (6)	42	26 (4)	93
Trail making A	Speed (seconds)	47 (26)	48	56 (36)	32	50 (36)	80
Trail making B	Speed (seconds)	130 (80)	48	221 (173)	22	142 (113)	70
Complex rey figure (copy)	Total score (out of 36)	34 (3)	37	31 (4)	21	34 (4)	58
Complex rey figure organizational strategy score	Total score (out of 6)	3.5 (3)	36	2 (1)	20	3 (3)	56
Complex rey figure delayed	Total correct (out of 36)	10 (8)	27	4 (8)	13	7.5 (8)	40
Forward digit span	Total score	6 (2)	43	5 (1)	31	6 (2)	74
Backward digit span	Total score	4 (1)	43	4 (1)	31	4 (2)	74
Stroop card 1	Reaction time (seconds)	46 (11)	37	54 (19)	28	49 (14)	65
Stroop card 2	Reaction time (seconds)	62 (16)	37	75 (33)	28	68 (22)	65
Stroop card 3	Reaction time (seconds)	118 (42)	37	158 (79)	24	131 (54)	61
15 words test delayed	Total score	3 (2)	43	0 (1)	30	1 (3)	73
Category fluency (animals)	Total score in 60 seconds	18 (6)	45	13 (5)	33	16 (7)	78

MCI = Mild Cognitive Impairment; AD = (dementia due to) Alzheimer’s Disease; MMSE = Mini Mental State Examination

Table S2. Neuropsychological test results of trial participants at baseline (n=55). Unstandardized scores are presented (median with interquartile range). The detection, identification, one-back and one card learning tests are part of the Cogstate Brief Battery™ (Maruff et al., 2013).

Neuropsychological assessment	Primary outcome measure	MCI (n=37)	AD (n=18)	Total
MMSE	Total score (out of 30)	27 (4)	24 (3)	26 (5)
Detection test	Speed ($\log_{10}$ milliseconds)	2.5 (0.2)	2.5 (0.2)	2.5 (0.1)
Identification test	Speed ($\log_{10}$ milliseconds)	2.7 (0.1)	2.7 (0.1)	2.7 (0.1)
One back test	Speed ($\log_{10}$ milliseconds)	3.0 (0.1)	3.1 (0.1)	3.0 (0.1)
One card learning test	Proportion correct (arcsine square root)	0.85 (0.1)	0.83 (0.1)	0.85 (0.1)

MCI = Mild Cognitive Impairment; AD = (dementia due to) Alzheimer's Disease; MMSE = Mini Mental State Examination

Table S3. Comparison of baseline EEG measures between diagnostic groups.

	MCI	AD	Total	P-value
Global (mean, SD)				
Theta power	0.145 (0.066)	0.181 (0.087)	0.160 (0.077)	**<.01****
Alpha1 power	0.187 (0.120)	0.143 (0.098)	0.169 (0.113)	**<.05***
Alpha2 power	0.122 (0.065)	0.113 (0.077)	0.118 (0.070)	0.10
Beta power	0.185 (0.073)	0.163 (0.074)	0.176 (0.074)	0.06
AEC-c alpha	0.546 (0.033)	0.540 (0.029)	0.544 (0.031)	0.24
AEC-c beta	0.526 (0.012)	0.524 (0.011)	0.525 (0.012)	0.46
PLI theta	0.150 (0.038)	0.144 (0.024)	0.148 (0.033)	0.96
PE theta	0.385 (0.027)	0.381 (0.027)	0.384 (0.027)	0.29
wSMI theta	0.182 (0.021)	0.179 (0.019)	0.181 (0.020)	0.50
JPE_{inv} theta	0.429 (0.023)	0.434 (0.019)	0.431 (0.022)	0.31
Parieto-occipital (mean, SD)				
Peak-frequency	9.28 (1.10)	8.86 (1.35)	9.11 (1.22)	0.11

MCI = Mild Cognitive Impairment; AD = (dementia due to) Alzheimer's Disease; SD = Standard Deviation; AEC-c = corrected Amplitude Envelope Correlation; PLI = Phase Lag Index; wSMI = weighted Symbolic Mutual Information; PE = Permutation Entropy; JPE_{inv} = inverted Joint Permutation Entropy.

Table S4. Results of the group-wise LMM analysis performed to evaluate change in parieto-occipital EEG measures over time (*in months*) in Aβ+ MCI or AD dementia patients. Crude and adjusted regression coefficients (β) and 95% confidence intervals (CI) are reported.

Region	Measure	Frequency band		Predictor	β	95% CI		p-value	Adjusted β[a]	95% CI[a]		p-value[a]
Parieto-occipital	Relative power	Theta[2]	MCI	*Time*	1.6E-03	8.3E-04	2.3E-03	**<.001*** **	1.8E-03	9.6E-04	2.7E-03	**<.001*** **
			AD	*Time*	8.7E-04	-2.0E-04	1.9E-03	0.12	1.1E-03	-5.0E-05	2.2E-03	0.06
			MCI-AD	*Time x Group*	9.0E-04	-4.7E-04	2.3E-03	0.20	7.6E-04	-6.6E-04	2.2E-03	0.29
		Alpha1[1]	MCI	*Time*	3.8E-04	-1.1E-03	1.9E-03	0.62	2.9E-04	-1.2E-03	1.8E-03	0.70
			AD	*Time*	-1.2E-03	-3.0E-03	5.2E-04	0.17	-1.3E-03	-3.2E-03	5.5E-04	0.17
			MCI-AD	*Time x Group*	1.6E-03	-7.0E-04	3.9E-03	0.17	1.6E-03	-7.7E-04	4.0E-03	0.18
		Alpha2[1]	MCI	*Time*	-1.3E-04	-1.5E-03	1.2E-03	0.86	-1.7E-04	-1.5E-03	1.2E-03	0.81
			AD	*Time*	-4.5E-04	-2.0E-03	1.1E-03	0.58	-3.4E-04	-2.0E-03	1.4E-03	0.69
			MCI-AD	*Time x Group*	3.2E-04	-1.8E-03	2.4E-03	0.76	1.7E-04	-2.0E-03	2.3E-03	0.87
		Beta[1]	MCI	*Time*	-1.0E-03	-1.8E-03	-2.7E-04	**<.01** **	-1.1E-03	-1.9E-03	-3.0E-04	**<.01** **
			AD	*Time*	-5.0E-04	-1.4E-03	3.9E-04	0.27	-6.7E-04	-1.6E-03	2.9E-04	0.17
			MCI-AD	*Time x Group*	-5.4E-04	-1.7E-03	6.4E-04	0.36	-4.0E-04	-1.6E-03	8.3E-04	0.52
	Peak-frequency[1]	-	MCI	*Time*	-1.0E-02	-2.1E-02	1.2E-03	0.08	-1.1E-02	-2.3E-02	-1.5E-06	**<.05***
			AD	*Time*	-2.3E-02	-3.6E-02	-9.6E-03	**<.001*** **	-2.3E-02	-3.7E-02	-9.2E-03	**<.01** **
			MCI-AD	*Time x Group*	1.3E-02	-4.5E-03	3.0E-02	0.15	1.2E-02	-5.9E-03	3.0E-02	0.19
	AEC-c	Alpha[1]	MCI	*Time*	-1.5E-04	-6.1E-04	3.1E-04	0.53	-1.6E-04	-6.2E-04	3.0E-04	0.48
			AD	*Time*	2.1E-05	-5.1E-04	5.5E-04	0.94	1.2E-04	-4.4E-04	6.9E-04	0.68
			MCI-AD	*Time x Group*	-1.7E-04	-8.7E-04	5.3E-04	0.64	-2.9E-04	-1.0E-03	4.4E-04	0.44
		Beta[1]	MCI	*Time*	-7.1E-05	-2.4E-04	9.9E-05	0.41	-6.7E-05	-2.4E-04	1.0E-04	0.43
			AD	*Time*	-1.2E-04	-3.2E-04	7.5E-05	0.22	-1.6E-04	-3.7E-04	5.1E-05	0.14
			MCI-AD	*Time x Group*	5.2E-05	-2.1E-04	3.1E-04	0.69	9.0E-05	-1.8E-04	3.6E-04	0.51
	PLI	Theta[1]	MCI	*Time*	1.9E-04	-3.4E-04	7.2E-04	0.48	1.8E-04	-3.5E-04	7.1E-04	0.50

		AD	*Time*	2.1E-04	-4.1E-04	8.3E-04	0.50	1.5E-04	-5.1E-04	8.0E-04	0.65
		MCI-AD	*Time x Group*	-2.1E-05	-8.3E-04	7.9E-04	0.96	3.2E-05	-8.1E-04	8.7E-04	0.94
PE	Theta[1]	MCI	*Time*	-2.4E-05	-2.5E-04	2.0E-04	0.84	-4.4E-05	-2.7E-04	1.8E-04	0.71
		AD	*Time*	-2.0E-04	-4.6E-04	6.4E-05	0.14	-1.5E-04	-4.4E-04	1.3E-04	0.29
		MCI-AD	*Time x Group*	1.7E-04	-1.7E-04	5.2E-04	0.32	1.1E-04	-2.5E-04	4.7E-04	0.55
wSMI	Theta[1]	MCI	*Time*	-1.8E-05	-1.9E-04	1.5E-04	0.83	-2.8E-05	-2.0E-04	1.4E-04	0.74
		AD	*Time*	1.4E-04	-5.2E-05	3.4E-04	0.15	7.9E-05	-1.3E-04	2.9E-04	0.46
		MCI-AD	*Time x Group*	-1.6E-04	-4.2E-04	9.5E-05	0.21	-1.1E-04	-3.7E-04	1.6E-04	0.43
JPE_{inv}	Theta[1]	MCI	*Time*	7.6E-07	-1.4E-04	1.4E-04	0.99	1.6E-05	-1.2E-04	1.6E-04	0.82
		AD	*Time*	-1.3E-04	-2.9E-04	3.6E-05	0.13	-5.4E-05	-2.3E-04	1.2E-04	0.55
		MCI-AD	*Time x Group*	1.3E-04	-8.6E-05	3.4E-04	0.24	7.0E-05	-1.5E-04	2.9E-04	0.53

[a.] Adjusted for age, sex and medication use.

[1.] Random intercept on subject level.

[2.] Random intercept and random slope on subject level.

MCI = Mild Cognitive Impairment; AD = (dementia due to) Alzheimer’s Disease; SD = Standard Deviation; AEC-c = corrected Amplitude Envelope Correlation; PLI = Phase Lag Index; wSMI = weighted Symbolic Mutual Information; PE = Permutation Entropy; JPE_{inv} = inverted Joint Permutation Entropy.

Table S5. Results of the group-wise LMM analysis performed to evaluate change in temporal EEG measures over time (*in months*) in Aβ+ MCI or AD dementia patients. Crude and adjusted regression coefficients (β) and 95% confidence intervals (CI) are reported.

Region	Measure	Frequency band		Predictor	β	95% CI		p-value	Adjusted β[a]	95% CI[a]		p-value[a]
Temporal	Relative power	Theta[2]	MCI	*Time*	2.2E-03	1.2E-03	3.1E-03	**<.001***	2.2E-03	1.2E-03	3.1E-03	**<.001***
			AD	*Time*	8.2E-04	-3.1E-04	2.0E-03	0.16	8.1E-04	-3.9E-04	2.1E-03	0.18
			MCI-AD	*Time x Group*	1.3E-03	-1.3E-04	2.8E-03	0.08	1.4E-03	-1.9E-04	2.8E-03	0.08
		Alpha1[2]	MCI	*Time*	-3.7E-04	-1.3E-03	5.6E-04	0.44	-3.8E-04	-1.3E-03	6.2E-04	0.44
			AD	*Time*	-6.0E-04	-1.7E-03	5.0E-04	0.30	-7.3E-04	-1.9E-03	5.3E-04	0.24
			MCI-AD	*Time x Group*	2.2E-04	-1.2E-03	1.7E-03	0.76	3.6E-04	-1.2E-03	1.9E-03	0.65
		Alpha2[1]	MCI	*Time*	-2.5E-04	-8.4E-04	3.4E-04	0.41	-2.9E-04	-8.8E-04	3.0E-04	0.34
			AD	*Time*	-5.7E-04	-1.3E-03	1.1E-04	0.10	-5.3E-04	-1.3E-03	2.1E-04	0.16
			MCI-AD	*Time x Group*	3.3E-04	-5.8E-04	1.2E-03	0.48	2.3E-04	-7.1E-04	1.2E-03	0.62
		Beta[1]	MCI	*Time*	-8.0E-04	-1.4E-03	-1.8E-04	**<.05***	-8.5E-04	-1.5E-03	-2.2E-04	**<.01****
			AD	*Time*	-5.8E-04	-1.3E-03	1.5E-04	0.12	-6.6E-04	-1.4E-03	1.2E-04	0.10
			MCI-AD	*Time x Group*	-2.2E-04	-1.2E-03	7.3E-04	0.64	-1.8E-04	-1.2E-03	8.1E-04	0.72
	AEC-c	Alpha[1]	MCI	*Time*	-1.6E-04	-5.8E-04	2.7E-04	0.47	-1.6E-04	-5.8E-04	2.7E-04	0.47
			AD	*Time*	-2.2E-05	-5.1E-04	4.7E-04	0.93	8.4E-05	-4.4E-04	6.1E-04	0.75
			MCI-AD	*Time x Group*	-1.3E-04	-7.8E-04	5.1E-04	0.68	-2.4E-04	-9.1E-04	4.3E-04	0.48
		Beta[1]	MCI	*Time*	-2.5E-05	-1.9E-04	1.4E-04	0.77	-2.4E-05	-1.9E-04	1.4E-04	0.78
			AD	*Time*	-5.2E-05	-2.5E-04	1.4E-04	0.60	-1.0E-04	-3.1E-04	1.0E-04	0.33
			MCI-AD	*Time x Group*	2.8E-05	-2.3E-04	2.9E-04	0.83	8.0E-05	-1.9E-04	3.5E-04	0.55
	PLI	Theta[1]	MCI	*Time*	1.1E-04	-3.7E-04	5.9E-04	0.66	1.1E-04	-3.8E-04	5.9E-04	0.67
			AD	*Time*	1.5E-04	-4.0E-04	7.1E-04	0.59	1.5E-04	-4.4E-04	7.4E-04	0.61
			MCI-AD	*Time x Group*	-4.8E-05	-7.8E-04	6.9E-04	0.90	-4.8E-05	-8.1E-04	7.1E-04	0.90
	PE	Theta[1]	MCI	*Time*	-9.2E-05	-3.0E-04	1.2E-04	0.39	-1.1E-04	-3.1E-04	1.0E-04	0.32

		AD	*Time*	-2.5E-04	-4.9E-04	-7.6E-06	**<.05***	-2.3E-04	-4.9E-04	3.9E-05	0.09
		MCI-AD	*Time x Group*	1.6E-04	-1.6E-04	4.8E-04	0.33	1.2E-04	-2.1E-04	4.5E-04	0.49
wSMI	Theta[1]	MCI	*Time*	1.5E-05	-1.4E-04	1.7E-04	0.85	7.6E-06	-1.5E-04	1.7E-04	0.93
		AD	*Time*	1.5E-04	-2.8E-05	3.4E-04	0.10	1.0E-04	-9.3E-05	3.0E-04	0.30
		MCI-AD	*Time x Group*	-1.4E-04	-3.8E-04	1.0E-04	0.25	-9.7E-05	-3.5E-04	1.5E-04	0.44
JPE_{inv}	Theta[1]	MCI	*Time*	-5.0E-05	-1.7E-04	6.7E-05	0.40	-9.7E-05	-2.2E-04	2.7E-05	0.13
		AD	*Time*	-1.3E-04	-2.7E-04	6.9E-06	0.07	-1.5E-04	-3.0E-04	8.5E-06	0.07
		MCI-AD	*Time x Group*	8.0E-05	-1.0E-04	2.6E-04	0.38	4.9E-05	-1.4E-04	2.4E-04	0.61

[a.] Adjusted for age, sex and medication use.

[1.] Random intercept on subject level.

[2.] Random intercept and random slope on subject level.

MCI = Mild Cognitive Impairment; AD = (dementia due to) Alzheimer's Disease; SD = Standard Deviation; AEC-c = corrected Amplitude Envelope Correlation; PLI = Phase Lag Index; wSMI = weighted Symbolic Mutual Information; PE = Permutation Entropy; JPE_{inv} = inverted Joint Permutation Entropy.

Table S6. Results of the group-wise LMM analysis performed to evaluate change in global EEG measures over time (*in months*) in Aβ+ MCI or AD dementia patients. Crude and adjusted regression coefficients (β) and 95% confidence intervals (CI) are reported.

Region	Measure	Frequency band		Predictor	β	95% CI		p-value	Adjusted β[a]	95% CI[a]		p-value[a]
Global	Relative power	Theta[2]	MCI	*Time*	1.8E-03	1.1E-03	2.6E-03	**<.001*** **	1.9E-03	1.1E-03	2.7E-03	**<.001*** **
			AD	*Time*	9.3E-04	-4.6E-05	1.9E-03	0.07	1.1E-03	3.5E-05	2.1E-03	**<.05***
			MCI-AD	*Time x Group*	9.0E-04	-3.5E-04	2.1E-03	0.16	8.2E-04	-4.8E-04	2.1E-03	0.21
		Alpha1[2]	MCI	*Time*	-2.4E-04	-1.3E-03	7.8E-04	0.64	-2.5E-04	-1.3E-03	8.5E-04	0.63
			AD	*Time*	-9.9E-04	-2.2E-03	1.8E-04	0.10	-1.1E-03	-2.4E-03	2.7E-04	0.11
			MCI-AD	*Time x Group*	7.5E-04	-8.0E-04	2.3E-03	0.35	8.2E-04	-8.4E-04	2.5E-03	0.33
		Alpha2[1]	MCI	*Time*	-1.9E-04	1.0E-03	6.2E-04	0.64	-2.5E-04	-1.1E-03	5.6E-04	0.55
			AD	*Time*	-4.8E-04	-1.4E-03	4.6E-04	0.32	-4.4E-04	-1.4E-03	5.6E-04	0.39
			MCI-AD	*Time x Group*	2.9E-04	-9.5E-04	1.5E-03	0.65	1.9E-04	-1.1E-03	1.5E-03	0.77
		Beta[1]	MCI	*Time*	-7.0E-04	-1.3E-03	-5.1E-05	**<.05***	-7.6E-04	-1.4E-03	-9.8E-05	**<.05***
			AD	*Time*	-3.2E-04	-1.1E-03	4.3E-04	0.40	-4.8E-04	-1.3E-03	3.4E-04	0.25
			MCI-AD	*Time x Group*	-3.8E-04	-1.4E-03	6.2E-04	0.46	-2.8E-04	-1.3E-03	7.6E-04	0.60
	AEC-c	Alpha[1]	MCI	*Time*	-1.7E-04	-5.9E-04	2.5E-04	0.42	-1.8E-04	-6.0E-04	2.4E-04	0.40
			AD	*Time*	1.3E-05	-4.7E-04	5.0E-04	0.96	1.3E-04	-3.9E-04	6.4E-04	0.62
			MCI-AD	*Time x Group*	-1.8E-04	-8.3E-04	4.6E-04	0.57	-3.1E-04	-9.7E-04	3.5E-04	0.36
		Beta[1]	MCI	*Time*	-3.4E-05	-1.9E-04	1.2E-04	0.67	-3.3E-05	-1.9E-04	1.3E-04	0.68
			AD	*Time*	-9.1E-05	-2.7E-04	9.3E-05	0.33	-1.2E-04	-3.2E-04	7.1E-05	0.21
			MCI-AD	*Time x Group*	5.7E-05	-1.9E-04	3.0E-04	0.65	9.1E-05	-1.6E-04	3.4E-04	0.48
	PLI	Theta[1]	MCI	*Time*	1.3E-04	-3.2E-04	5.8E-04	0.58	1.3E-04	-3.3E-04	5.8E-04	0.58
			AD	*Time*	1.8E-04	-3.5E-04	7.0E-04	0.50	1.9E-04	-3.6E-04	7.5E-04	0.50
			MCI-AD	*Time x Group*	-5.0E-05	-7.4E-04	6.4E-04	0.89	-6.5E-05	-7.8E-04	6.5E-04	0.86
	PE	Theta[1]	MCI	*Time*	-5.5E-05	-2.6E-04	1.5E-04	0.60	-7.6E-05	-2.8E-04	1.3E-04	0.47

		AD	*Time*	-2.3E-04	-4.6E-04	1.2E-05	0.06	-1.9E-04	-4.5E-04	6.8E-05	0.18
		MCI-AD	*Time x Group*	1.7E-04	-1.4E-04	4.8E-04	0.28	1.2E-04	-2.1E-04	4.4E-04	0.49
wSMI	Theta[1]	MCI	*Time*	2.9E-06	-1.5E-04	1.6E-04	0.97	-4.8E-06	-1.6E-04	1.5E-04	0.95
		AD	*Time*	1.5E-04	-3.3E-05	3.3E-04	0.11	9.7E-05	-1.0E-04	2.9E-04	0.34
		MCI-AD	*Time x Group*	-1.5E-04	-3.9E-04	9.3E-05	0.23	-1.0E-04	-3.5E-04	1.5E-04	0.42
JPE_{inv}	Theta[1]	MCI	*Time*	-5.6E-06	-1.4E-04	1.3E-04	0.93	1.2E-05	-1.2E-04	1.5E-04	0.86
		AD	*Time*	-1.2E-04	-2.8E-04	3.4E-05	0.12	-5.6E-05	-2.3E-04	1.2E-04	0.52
		MCI-AD	*Time x Group*	1.2E-04	-8.8E-05	3.2E-04	0.26	6.7E-05	-1.5E-04	2.8E-04	0.53

[a.] Adjusted for age, sex and medication use.

[1.] Random intercept on subject level.

[2.] Random intercept and random slope on subject level.

MCI = Mild Cognitive Impairment; AD = (dementia due to) Alzheimer's Disease; SD = Standard Deviation; AEC-c = corrected Amplitude Envelope Correlation; PLI = Phase Lag Index; wSMI = weighted Symbolic Mutual Information; PE = Permutation Entropy; JPE_{inv} = inverted Joint Permutation Entropy.

Table S7. Longitudinal effect sizes of whole-group LMM results.

Analyzed subjects	Region	Frequency band	Measure	Effect size (50% less deterioration)		Effect size (stabilization)[3]		Effect size (50% improvement)	
				1 year	2 years	1 year	2 years	1 year	2 years
Whole-group (MCI + AD dementia)	Temporal	Theta[2]	Relative power	0.11	0.23	0.23	0.45	0.34	0.68
	Parieto-occipital[1]	-	Peak frequency	0.07	0.15	0.15	0.30	0.22	0.45
	Temporal	Beta[1]	Relative power	0.06	0.12	0.12	0.24	0.18	0.35

[1] Random intercept on subject level
[2] Random intercept and random slope on subject level.
[3]Effect sizes as directly computed from the LMM results; magnitude of the natural change of the EEG outcome measures over time.
MCI = Mild Cognitive Impairment; AD = (dementia due to) Alzheimer's Disease; PE = Permutation Entropy

Table S8. Longitudinal effect sizes of group-wise LMM results.

Analyzed subjects	Region	Frequency band	Measure	Effect size (50% less deterioration)		Effect size (stabilization)[3]		Effect size (50% improvement)	
				1 year	2 years	1 year	2 years	1 year	2 years
MCI	Temporal	Theta[2]	Relative power	0.15	0.30	0.30	0.60	0.45	0.89
	Parieto-occipital	Beta[1]	Relative power	0.07	0.14	0.14	0.28	0.21	0.42
AD dementia	Parieto-occipital[1]	-	Peak frequency	0.11	0.23	0.23	0.45	0.34	0.68
	Temporal	Theta[1]	PE	0.06	0.11	0.11	0.22	0.17	0.33

[1] Random intercept on subject level
[2] Random intercept and random slope on subject level.
[3]Effect size as directly computed from the LMM results; magnitude of the natural change of the EEG outcome measures over time.
MCI = Mild Cognitive Impairment; AD = (dementia due to) Alzheimer's Disease; PE = Permutation Entropy

Table S9. Three-month effect sizes (measured as Cohen's *d*) for EEG measures that demonstrated a significant change over time in the trial dataset (n=55, median follow-up duration 3 months), clinical dataset (n=93, median follow-up duration 13.2 months) and total dataset (n=148, median follow-up duration 7.2 months). In the trial dataset, only relative theta power exhibited a significant change over time. In the clinical dataset, additional measures (i.e., relative *beta* power, peak frequency and permutation entropy) demonstrated significant change over time. Effect sizes in the trial dataset are considerably larger than those in the clinical dataset. When considering the results of the full dataset, they align more closely with the clinical data.

Cohort	Region	Frequency band	Measure	d (3 months)
Trial	Parieto-occipital	Theta	Relative power	0.16
	Whole-brain	Theta	Relative power	0.15
	Temporal	Theta	Relative power	0.13
Clinical	Temporal	Theta	Relative power	0.05
	Whole-brain	Theta	Relative power	0.05
	Parieto-occipital	Theta	Relative power	0.03
	Parieto-occipital	-	Peak frequency	0.03
	Temporal	Beta	Relative power	0.03
	Parieto-occipital	Beta	Relative power	0.03
	Whole-brain	Beta	Relative power	0.02
	Temporal	Theta	Permutation entropy	0.02
Total	Temporal	Theta	Relative power	0.06
	Whole-brain	Theta	Relative power	0.06
	Parieto-occipital	Theta	Relative power	0.05
	Parieto-occipital	-	Peak frequency	0.04
	Parieto-occipital	Beta	Relative power	0.03
	Temporal	Beta	Relative power	0.03
	Whole-brain	Beta	Relative power	0.02
